# Unraveling the Metabolic Derangements Occurring in Non-infarcted Areas of Pig Hearts With Chronic Heart Failure

**DOI:** 10.3389/fcvm.2021.753470

**Published:** 2021-10-13

**Authors:** Cláudia Correia, Qing-Dong Wang, Gunilla Linhardt, Leif G. Carlsson, Benjamin Ulfenborg, Anna Walentinsson, Katarina Rydén-Markinhutha, Margareta Behrendt, Johannes Wikström, Peter Sartipy, Karin Jennbacken, Jane Synnergren

**Affiliations:** ^1^Systems Biology Research Center, Translational Bioinformatics Research Group, School of Biosciences, University of Skövde, Skövde, Sweden; ^2^Bioscience Cardiovascular, Research and Early Development, Cardiovascular, Renal and Metabolism (CVRM), BioPharmaceuticals R&D, AstraZeneca, Gothenburg, Sweden; ^3^Translational Science & Experimental Medicine, Research and Early Development, Cardiovascular, Renal and Metabolism (CVRM), BioPharmaceuticals R&D, AstraZeneca, Gothenburg, Sweden; ^4^Late-Stage Development, Cardiovascular, Renal and Metabolism (CVRM), BioPharmaceuticals R&D, AstraZeneca, Gothenburg, Sweden

**Keywords:** chronic heart failure, transcriptome (RNA-seq), metabolome, myocardial infarction (MI), decompensated heart failure

## Abstract

**Objective:** After myocardial infarction (MI), the non-infarcted left ventricle (LV) ensures appropriate contractile function of the heart. Metabolic disturbance in this region greatly exacerbates post-MI heart failure (HF) pathology. This study aimed to provide a comprehensive understanding of the metabolic derangements occurring in the non-infarcted LV that could trigger cardiovascular deterioration.

**Methods and Results:** We used a pig model that progressed into chronic HF over 3 months following MI induction. Integrated gene and metabolite signatures revealed region-specific perturbations in amino acid- and lipid metabolism, insulin signaling and, oxidative stress response. Remote LV, in particular, showed impaired glutamine and arginine metabolism, altered synthesis of lipids, glucose metabolism disorder, and increased insulin resistance. *LPIN1, PPP1R3C, PTPN1, CREM*, and *NR0B2* were identified as the main effectors in metabolism dysregulation in the remote zone and were found differentially expressed also in the myocardium of patients with ischemic and/or dilated cardiomyopathy. In addition, a simultaneous significant decrease in arginine levels and altered *PRCP, PTPN1*, and *ARF6* expression suggest alterations in vascular function in remote area.

**Conclusions:** This study unravels an array of dysregulated genes and metabolites putatively involved in maladaptive metabolic and vascular remodeling in the non-infarcted myocardium and may contribute to the development of more precise therapies to mitigate progression of chronic HF post-MI.

## Graphical Abstract

Summary of the metabolic and vascular derangements found in border and remote zones of pig hearts three months after the MI. 
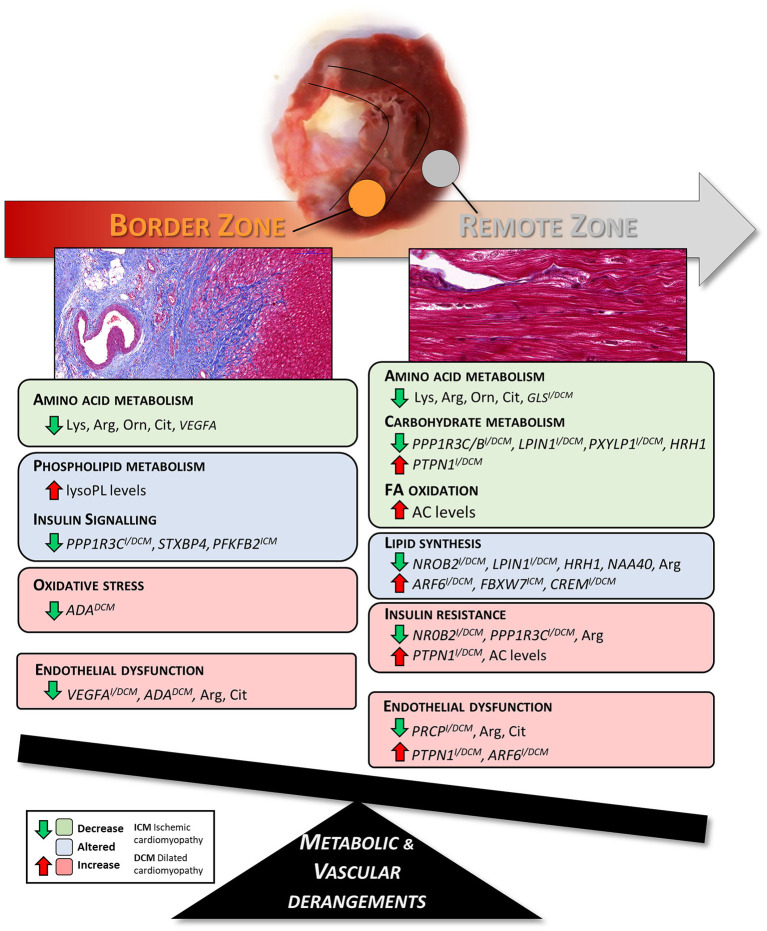

## Introduction

Recent advances in cardiovascular medicine have considerably improved heart failure (HF) management (1). More efficient reperfusion strategies, pharmacologic therapies, and implantable cardiac assist devices have significantly improved patients survival after acute myocardial infarction (MI) (2). In contrast, the burden of patients suffering from chronic HF as well as the occurrence of sudden cardiac death in post-MI patients considered clinically stable continue to increase worldwide. This occurs, mainly because HF is a silent progressive disease in which cardiac structure and function continue to deteriorate over time, often without revealing clear clinical evidence and symptoms of a worsening disease state (3). Thus, the deterioration process underlying chronic HF remains undertreated.

In fact, after MI, the heart undergoes an extremely dynamic and complex remodeling process that gradually progresses to a variety of cellular and subcellular maladaptations in the infarcted and non-infarcted areas of the myocardium (4). Several studies have investigated the structural and functional remodeling that occurs in the infarcted, border, and remote myocardium zones post-MI (5). It has been shown that these myocardium areas present differences in ionic currents (6), Ca^2+^ handling (7), and contractility (8) during the post-MI remodeling process. Some studies have reported region-specific myocardial gene- and/or protein expression patterns early after MI (9–11). Pavo et al., performed differential gene expression analysis of infarcted, border zone, and remote myocardium, 1 month after MI, in a reperfused MI porcine model to access the effect of percutaneously intramyocardially delivery of apoptotic peripheral blood cells secretome (12). Nonetheless, there is still a lack of detailed region-specific transcriptomic studies at later stages during the progression of HF, stages at which new therapeutic interventions are needed to improve HF patients' outcome.

To our knowledge, no studies have assessed the region-specific myocardial changes in metabolism after MI. It is well-known that during chronic HF, cardiac metabolism is perturbed in a chronic manner, resulting in impaired mitochondrial function and oxidative capacity, metabolic inflexibility, and energy depletion that contribute to the worsening of cardiac function. Nevertheless, it is still unclear how the metabolic dysregulation is triggered and which molecular pathways that are involved, specifically in non-infarcted areas. Clinical metabolomic profiling studies to improve HF diagnosis and/or prognosis have been carried out (13). However, due to limited access to heart biopsies, these studies have been restricted to measurements of plasma- and urine metabolites, which reveal only limited information about the metabolic changes occurring within the heart tissue. In-depth characterization of the myocardial region-specific metabolic derangements associated with HF progression in a translatable large animal model could be of major importance to identify biomarkers for HF progression and reveal potential targets for therapies capable to prevent and/or retard the progressive HF deterioration process. Characterization of the still healthy remote area from MI, would be of particular interest, potentially enabling to capture the first molecular alterations that occur in the transition from chronic compensated HF toward decompensated HF.

Herein, we performed an untargeted multi-omics integrative analysis to improve the understanding of region-specific remodeling at later stages post-MI. We used a translational pig MI model developing chronic HF over the course of 3 months, following a temporary occlusion of the left anterior descending coronary artery (LAD) (14), and systematically investigated the changes in the transcriptome- and metabolome profiles in the MI border and remote myocardium areas of pig hearts, aiming to identify targets that could trigger maladaptive remodeling. This model has been used for cardiovascular studies and for late preclinical testing of new cardiovascular drug candidates (14). There are several advantages with this pig model including: (1) similar cardiac anatomy and physiology to human, (2) larger size than most other animal species making it amenable to induce MI by intracoronary balloon occlusion, and (3) the time course of myocardium remodeling resembles that observed in humans (15). The use of this model limited the confounding variables (e.g., due to environment, comorbidities, different drug medications, and nutritional habits) typically present in human data.

In this study, we mapped the dysregulated genes and metabolites in the different left ventricular (LV) regions to relevant biological pathways and identified patterns of interrelated gene-metabolite-function changes. Our results showed perturbations in amino acid and lipid metabolism, altered insulin signaling and activation of the oxidative stress response in non-infarcted myocardial areas, and unveiled targets that may potentially contribute to the deleterious effects in chronic HF post-MI.

## Materials and Methods

### Induction of Myocardial Infarction

Six female Ellegaard Gottingen Minipigs (20–25 kg body weight, 1 year old) were used in this study. A catheter (Seldinger introducers) was placed in the right carotid artery for catheterization of the LAD. In the carotid artery introducer, a guide (GC Adroit, 6F, JR4, Johnson & Johnson, Sweden) were advanced to the aortic arch and by means of fluoroscopic guidance and injection of contrast agent (Visipaque 320, Apoteket, Sweden), the coronary vessels were visualized to enable positioning of a guide wire (PCI Wire, Wizdom SGW 0.014^′′^ 300 J Soft, Johnson & Johnson, Sweden or Choice Floppy guide wire H74) in the LAD. A balloon catheter (Maverick2 Monorail 9–12 mm × 3.5 mm) was advanced over the wire and positioned distal to the first diagonal branch of the LAD. To induce MI the balloon was inflated for 120 min (Pressure: 6–9 ATM). After 120 min of occlusion, the balloon was deflated to initiate reperfusion and the catheter was withdrawn. In case severe ventricular arrhythmias appeared, attempts to restore sinus rhythm by means of ventricular defibrillation and chest compression as well as injection of lidocaine were initiated. When electrically and hemodynamically stable, the skin was sutured together and the pigs brought back to the pen to wake up.

Three months after the MI procedure the pigs were sacrificed, the hearts excised and washed in chilled saline and chilled phosphate-buffered saline. Left ventricular free wall tissue samples (20 mg) were taken from the infarcted, border, and remote areas (few millimeters away from border zone) for subsequent transcriptomic- and metabolomic analysis and put in 2 mL tubes containing a cold steel bead and immediately frozen on dry ice, to assure fast freezing. Hearts were then weighed and sliced in 0.5 cm slices from apex to base for histological analysis. Control left ventricular free wall tissue was harvested from three naïve pigs not subjected to ischemia and reperfusion and following the same fast freezing procedure.

All animal procedures were undertaken according to the guidelines from Directive 2010/63/EU of the European Parliament on the protection of animals used for scientific purposes. The study was approved by the local Ethical Committee in Gothenburg, Sweden, 2016-06-29 (permit no 68 2016). Detailed information regarding the anesthetic agents used, the dose, and the route and frequency of administration can be found in Supplementary Data.

### Histological Analysis

Excised hearts were transversely sliced in 5 mm and fixated in formaldehyde. Subsequently, the slices were embedded in paraffin and further sectioned into 4 μm slices. Masson's Trichrome (MTC) staining was used to distinguish fibrotic areas from healthy cardiac tissue.

All immunohistochemistry methods and protocols were set up on the Ventana Discovery Ultra autostainer. Immunohistochemistry for detection of CD31, CD45, Wilm's tumor protein (WT1), and Myeloperoxidase (MPO) were carried out according to manufactures recommendation and all reagents except antibodies were Ventana products (Roche, Basel, Switzerland). Antigen retrieval was done in Ventana Cell Conditioner 1 for 40 min at 95°C. Primary antibody was added for 1 h at 37° followed by Antibody Block and secondary anti-rabbit HQ reagent and anti-HQ HRP, purple chromogenic detection. The following antibodies and dilutions were used: CD31 (dilution 1:50, ab28364, Abcam), CD45 (dilution 1:700, ab10558, Abcam), WT1 (dilution 1:20, ab89901, Abcam), and MPO (dilution 1:500, A0398, Dako).

### Assessment of Left Ventricular Function

Left ventricular function was assessed by echocardiography using a Philips EPIQ7G ultrasound instrument with a sector probe X5-1 (5–1 MHz) and the adult cardiac echo software version 1.5.1. The assessment was carried out under isoflurane inhalation anesthesia immediately before the MI surgery and 3 months post-MI while the pigs were positioned on either the right or the left side. Stroke volume (SV) was calculated as the difference between the left ventricular end-diastolic volume (LVEDV) and the left ventricular end-systolic volume (LVESV) and the left ventricular ejection fraction (LVEF) was calculated as the ratio between SV and LVEDV. Cardiac output (CO) was obtained from the product of heart rate (HR) and the SV and cardiac index describes the CO corrected for the body surface area (BSA). Regional left ventricular contractility were also assessed using Fractional Area Change (FAC) at the basal, midventricular, and apical level. The left ventricular area was traced in end-diastole and end-systole in PSAX views. The FAC formula used was (LVEDA-LVESA/LVEDA). Left ventricular deformation were evaluated with Longitudinal Strain (LS) in a modified Apical 4 chamber (A4C) view.

### Transcriptomics Analysis

Total RNA was isolated using RNeasy^®^ Fibrous Tissue Mini Kit (Qiagen).

RNA sequencing was performed at the National Genomic Infrastructure (NGI), Stockholm, Sweden. Library construction was performed using Illumina Truseq stranded total RNA with Illumina Ribozero method. Clustering was done by “cBot” and samples were sequenced on NovaSeq6000 (NovaSeq Control Software 1.6.0/RTA v3.4.4) with a 2 × 51 setup using “NovaSeqXp” workflow in “S1” mode flowcell. The Bcl to FastQ conversion was performed using bcl2fastq_v2.19.1.403 from the CASAVA software suite. The quality scale used was Sanger/phred33/Illumina 1.8+.

Sequenced reads were quality controlled with the FastQC software and pre-processed with Trim Galore. Processed reads were then aligned to the reference genome of Pig (*Sus scrofa*, Sscrofa10.2) with the STAR aligner. Read counts for genes were generated using the feature Counts library and normalized FPKM values were calculated with StringTie. The RNA-sequencing data are available at ArrayExpress (https://www.ebi.ac.uk/arrayexpress/experiments/E-MTAB-8856/).

### Data Analysis

Differentially expressed genes (DEGs) between all LV tissue areas [Infarcted Zone (IZ), Border Zone (BZ), and Remote Zone (RZ)] and healthy LV control tissue were determined using the Bioconductor package edgeR and a combined criteria of FDR <0.05 and fold change FC ≥|1.5|. Principal component analysis (PCA) and hierarchical clustering were performed using standard functions in R version 3.5.0. Functional enrichment analysis of the DEGs was conducted using Gene Ontology (GO) resource (http://geneontology.org/), PANTHER database (http://www.pantherdb.org), and DAVID bioinformatic resource 6.8 (https://david.ncifcrf.gov). DEGs were also analyzed using QIAGEN's Ingenuity^®^ Pathway Analysis software (IPA^®^, QIAGEN, www.qiagen.com/ingenuity) to identify significantly dysregulated canonical pathways and biological functions. Functional network analysis was also performed using IPA as described in the Supplementary Material.

### Human Target Validation

Human heart-tissue level transcriptomics data from three publicly available independent studies (NCBI Gene Expression Omnibus database accession numbers GSE5406, GSE1145, and GSE65446) were used for comparison with the generated post-MI Pig dataset. Dataset GSE5406 compared 86 dilated cardiomyopathy (DCM), 108 ischemic cardiomyopathy (ICM) patients with 16 controls (16). Data set GSE1145 consists of 11 ICM, 15 DCM, and 11 controls (www.cardiogenomics.org). Dataset GSE65446 includes 6 DCM and 4 controls (17). More detailed information regarding these studies is provided in Supplementary Table 1.

Differential expression analyses (Disease vs. Normal) were conducted using QIAGEN's OmicSoft DiseaseLand (release HumanDisease_B37_20191215_v14a), using generalized linear models (glm) for log2 transformed intensities (microarray studies GSE1145 and GSE5406), and DESeq2 for raw counts data (RNAseq study GSE65446). Genes were considered significantly differentially expressed at FDR <0.05.

### Metabolomics Analysis

Metabolic profiling by GC-MS and LC-MS was performed at the Swedish Metabolomics Center in Umeå, Sweden. Information about reagents, solvents, standards, sample preparation, GC/LC-MS analysis, and metabolite identification can be found as Supplementary Material.

Mass spectra and retention index comparison was performed using NIST MS 2.0 software. Metabolites were identified with the HMDB database (http://hmdb.ca).

#### Data Analysis

To maximize identification of differences in metabolic profiles between the groups (BZ and RZ vs. healthy LV control tissue) and minimize other biological analytical variation, sample classes were modeled using the Orthogonal Projections to Latent Structures Discriminant Analysis (OPLS-DA) algorithm using the SIMCA-P software (version 15.0, Umetrics, Umeå, Sweden) after mean centering and unit variance scaling. The quality of the models is described by the R2X, R2Y, and Q2 values. R2X and R2Y indicate goodness of fit, and Q2 indicates predictability, calculated by a seven times cross-validation procedure. The following values were considered acceptable for good models predictability of metabolomics data: R2 >0.7, Q2 >0.4, and R2 not exceeding Q2 by more than 0.2 units (18) (SIMCA P12 users' guide, p514).

Metabolites with variable importance in the projection (VIP) values >1.2, determined in SIMCA, were considered as stronger contributors to discrimination among groups. Student's *t*-test was performed to further determine the statistical significance of the metabolic alterations between the groups using MetaboAnalyst 4.0 software. For this analysis, data were filtered using an interquartile range (IQR), log-transformed and auto-scaled. MetaboAnalyst 4.0, MBROLE 2.0, and KEGG database were used to identify the biochemical pathways linked to the identified metabolites.

### Statistical Analysis

The statistical analysis was performed in R (transcriptomics data), SIMCA/MetaboAnalyst (metabolomics data), and GraphPad Prism Software version 8 (Echocardiography data). Echocardiographic characteristics are represented as mean ± SEM of independent measurements and statistical significance was assessed using a paired Student's *t*-test. Correlation analysis between the normalized gene counts, the metabolite intensity levels, and the echocardiography measurements were analyzed using Spearman's correlation test in GraphPad. Values of *p* <0.05 were considered statistically significant.

## Results

### Development of Post-MI Pig Model With Chronic HF

**Figure 1:**
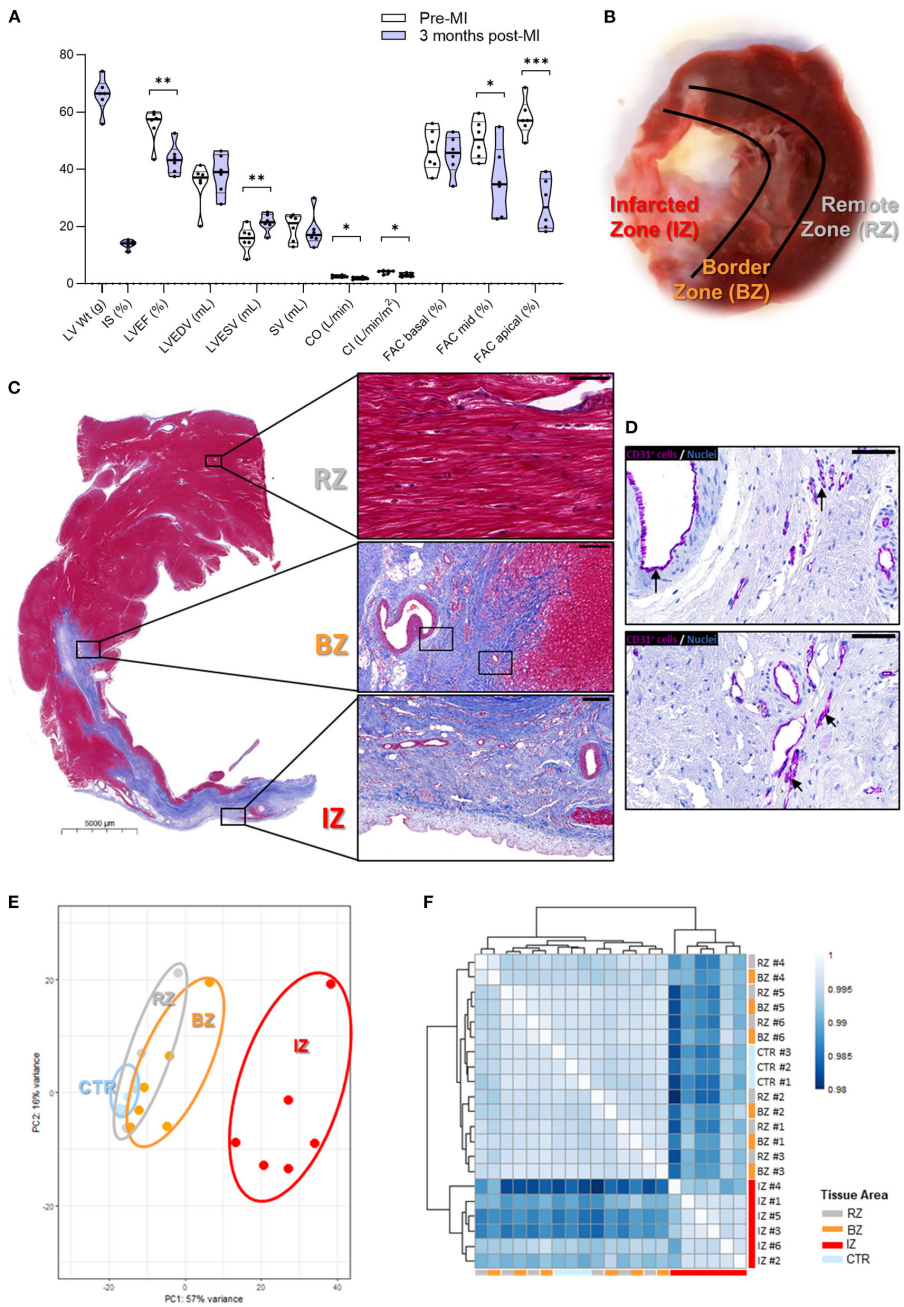
Gene expression profiling in different areas of chronic infarcted porcine hearts. **(A)** Global cardiac functional indices before, and 3 months after MI. LV Wt, left ventricular weight; IS, infarct size; LVEF, left ventricular ejection fraction; LVEDV, left ventricular end-diastolic volume; LVESV, left ventricular end systolic volume; SV, stroke volume; CO, cardiac output; CI, cardiac index; FAC, fractional area change. Data are shown as Violin plots with individual datapoints corresponding to each animal (*n* = 6). *P*-value from paired *t*-test. Contractile function data at baseline (pre-MI) and 3 months post-MI can be found in Supplementary Table 2. **(B)** Image of explanted pig heart showing an infarcted zone (IZ) distinguished by a pale region of scar tissue; the region directly adjacent to the infarct—termed border zone (BZ) and remote zone (RZ) of the myocardium. **(C)** Histological analysis showing: (i—upper panel) RZ with viable cardiac myocytes (scale bar: 50 μm); (ii—middle panel) BZ adjacent to IZ (scale bar: 200 μm); (iii—lower panel) IZ with necrotic and fibrotic tissue (scale bar: 200 μm). **(D)** CD31 staining in purple revealing the occurrence of neovessels in the IZ. Arrows highlight areas of positive CD31 staining. Scale bars: 50 μm. **(E)** Principal Component Analysis of the global transcriptome. **(F)** Sample distance heatmap and hierarchical clustering for all replicates. IZ/BZ/RZ: *n* = 6; control *n* = 3. ***P-value: 0.0001 to 0.001; **P-value: 0.001 to 0.01; *P-value: 0.01 to 0.05.

After developing HF for 3 months, the pigs exhibited an infarct size (IS) of ~15% of the LV mass and a significant 20% reduction in LVEF (Figure 1A and Supplementary Table 2). In addition, pigs showed a significant increase in the LVESV, a significant decrease in mid- and apical-region fractional area change, cardiac output, cardiac index, and longitudinal strain as well as and a trend of increased LVEDV and decreased SV, compared to the baseline levels (immediately before inducing the MI, Figure 1A and Supplementary Table 2). These results indicate a relevant extent of ischemic injury with clear reduction of systolic performance but slow progression to dilated remodeling, resembling the underlying process in HF progression typically observed in humans after MI. Images of the infarcted hearts and histological examination showed clear phenotypic changes between infarcted and non-infarcted LV areas (Figures 1B,C). In the infarcted area, cardiomyocytes were replaced by dense fibrotic tissue whereas in non-infarcted areas, cardiomyocytes maintained a normal morphology, and tissue architecture (Figure 1C). In the infarcted area, cells staining positive for the endothelial marker CD31 (Figure 1D), the hematopoietic lineage marker CD45 (Supplementary Figure 1A), and the neutrophil specific marker MPO (Supplementary Figure 1B) were detected, suggesting neovessel formation and infiltration of inflammatory cells.

### Region-Specific Remodeling of the Left Ventricle at Chronic HF Phase

To evaluate the transcriptional changes occurring at later stages after MI induction in different regions of the LV, RNA-sequencing analysis was performed in samples collected from the infarcted zone (IZ), from the border zone (BZ), and from a zone remotely from the infarct (RZ, Figures 1B,C). The gene expression in these specific LV areas was compared to control LV samples obtained from healthy animals. PCA (Figure 1E) and heatmap cluster analysis (Figure 1F) show that samples from the IZ demonstrate the largest transcriptional differences and clustered separately from the BZ and RZ samples. On the other hand, RZ and BZ clustered together and on the same branch as the control group (Figure 1F), suggesting a similar gene expression profile between the latter three groups of samples.

Pathway analysis of the differentially expressed genes (DEGs, FC >2, and FDR <0.05) in the IZ indicated that the significantly activated pathways are related to hypertrophy, apoptosis, fibrosis, and inflammation, whereas the repressed pathways represent mainly metabolic processes, related to energy production by oxidative phosphorylation [including TCA cycle, fatty acid oxidation (FAO), and acetyl-coA biosynthesis] and branched chain amino acid (BCAA) catabolism (Supplementary Figure 2A). These data corroborate the observed phenotypic changes in the IZ (Figures 1C,D), namely, the replacement of cardiomyocytes (rich in mitochondria and with high metabolic activity) by collagen, extracellular matrix proteins, and fibroblasts (with lower mitochondrial content and metabolic activity). Heatmaps of cell-population specific markers (Supplementary Figures 3A–E) confirm the decreased expression of some cardiomyocyte markers and increased expression of fibroblast, endothelial, mural and immune cells in the IZ. Counteracting this adverse remodeling of the LV, observed in the IZ, a few pathways and biological functions with a putative cardioprotective role were shown to be activated, including IGF1, NGF, HGF pathways, and angiogenesis/vasculogenesis functions (Supplementary Figures 2A,B). Notably, we observed an increase in the expression of genes involved in epicardium activation, endothelial-mesenchymal transition (EMT), and epicardium-derived cell (EPDC) differentiation (Supplementary Figure 3F), and detected infiltration of cells positive for the EPDC marker WT1 (by immunohistochemistry analysis) in the IZ (Supplementary Figures 1C,D). Since EPDCs may play an important role in the regenerative response to cardiac damage (19), these findings suggest that some cardiac healing still occurs in the IZ of the LV three months post-MI.

### Gene Expression Derangements in the Non-infarcted Areas of the Left Ventricle

To also consider genes with smaller but yet significant, and maybe biologically important changes in the RZ and BZ, a fold-change criteria of 1.5-fold (at FDR <0.05) was used for the analysis of the regions that are not directly affected by infarction. Using this selection criteria 66 DEGs were identified in the BZ and 104 DEGs in the RZ (Supplementary Table 3). Whereas, most of the DEGs in the BZ and RZ were down-regulated, slightly more DEGs were up-regulated in the IZ (Supplementary Figure 4).

**Figure 2:**
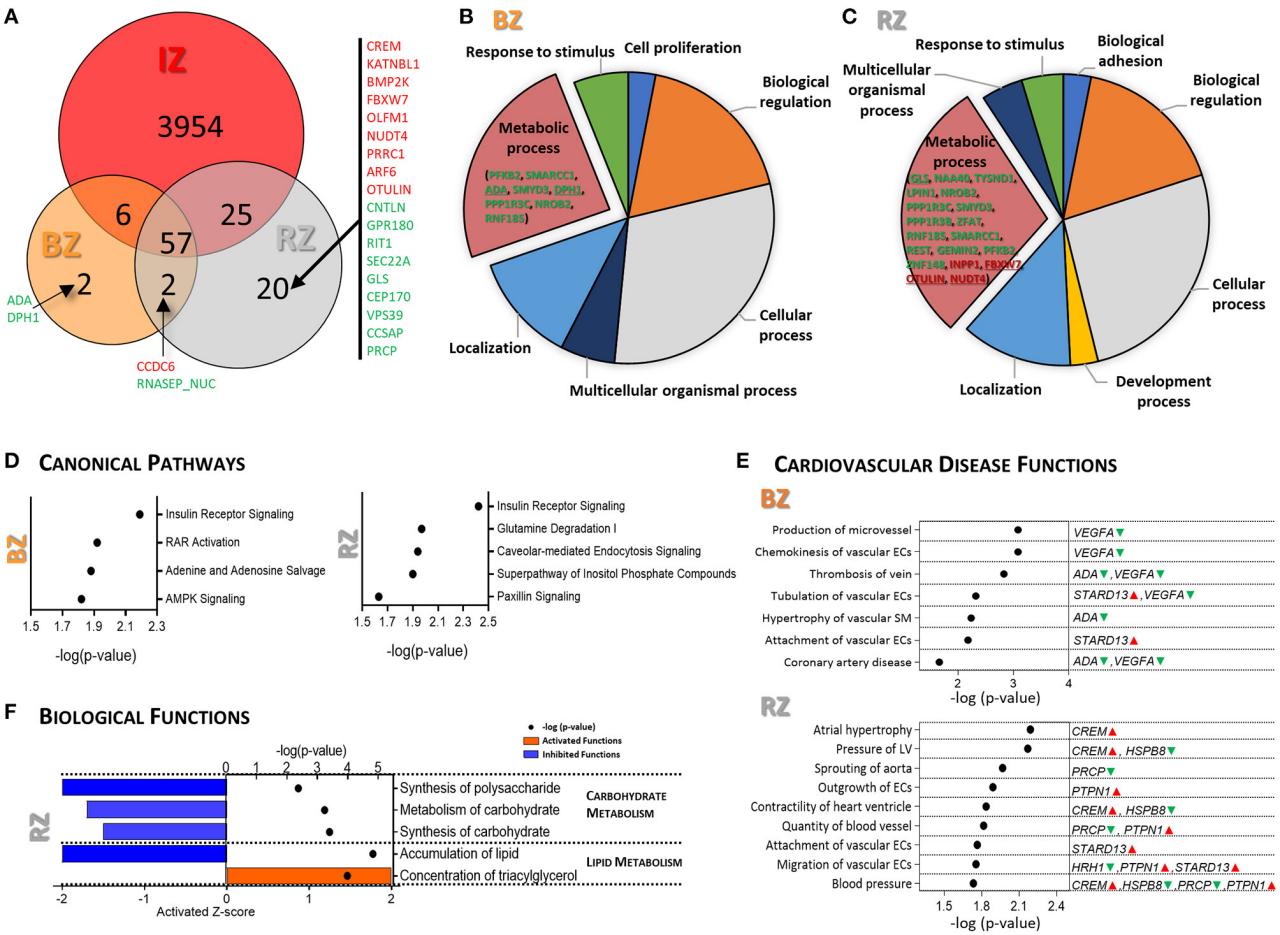
Gene expression analysis of Remote and Border tissue areas of infarcted pig hearts. **(A)** Venn diagram showing the overlapping DEGs (FC ≥|1.5| and FDR <0.05) between IZ, RZ and BZ. Genes marked in red are up-regulated and in green are down-regulated. Pie charts illustrating the results of the PANTHER GO-Slim biological process analysis for the DEGs in BZ **(B)** and RZ **(C)**. **(D)** IPA canonical pathway analysis for the DEGs in BZ (left) and RZ (right). *P*-value (right-tailed Fisher's exact test) <0.05. **(E)** Representative functional categories related to cardiovascular disease altered in the BZ (upper panel) and in the RZ (lower panel). Full list can be found in Supplementary Table 4. The genes/metabolites involved in each function are highlighted in green (down-regulated) and red (up-regulated). *P*-value (right-tailed Fisher's exact test) <0.05. **(F)** IPA biological functions predicted as activated (*Z*-score ≥1.5, orange) and inhibited (*Z*-score ≤ -1.5, blue) in RZ. *P*-value (right-tailed Fisher's exact test) <0.05. No activated/inhibited biological functions were identified in the BZ. IZ/BZ/RZ: *n* = 6; control *n* = 3.

Venn diagram shows that a total of 57 DEGs were found dysregulated in all three regions (RZ, BZ, and IZ); two DEGs (*DPH1* and *ADA*) were exclusively dysregulated in the BZ and 20 DEGs exclusively dysregulated in the RZ (Figure 2A). Gene ontology analysis indicated that the DEGs identified in the BZ were essentially connected to biological regulation, cellular and metabolic processes and the DEGs found in the RZ were largely involved in metabolic processes (Figures 2B,C).

Canonical pathway analysis for the DEGs in the BZ, revealed significant dysregulation of insulin receptor signaling, RAR activation, adenine and adenosine salvage and AMPK signaling (Figure 2D, left panel). The DEGs involved in these pathways are *PPP1R3C, STXBP4, SMARCC1, VEGFA, PFKFB2* and *ADA*, all of them were down-regulated in BZ comparing with control LV (Supplementary Table 4). The most significantly dysregulated cardiovascular related diseases and functions identified in the BZ are related with vascular endothelial and smooth muscle cells functional changes, and *VEGFA, ADA*, and *STARD13* are the main mediators (Figure 2E, upper panel).

Dysregulated canonical pathways (Figure 2D, right panel) and enriched biological functions (Figure 2F) in the RZ strongly point toward metabolic dysregulation at the gene expression level, indicating alterations not only in insulin signaling, as in the BZ, but also in glutamine degradation, carbohydrate-, and lipid metabolism and in the inositol phosphate pathway.

Glutamine degradation pathway was significantly dysregulated in the RZ (Figure 2D), mainly due to down-regulation of *GLS* exclusively in the RZ.

*PTPN1*, highly involved in insulin signaling and carbohydrate/lipid metabolism, was found to be significantly up-regulated in the RZ but not in the BZ, suggesting that *PTPN1* may have a key role in metabolic remodeling particularly in the RZ. Coordinated changes in gene expression in the RZ suggest a reduction in carbohydrate metabolism and altered lipid metabolism (Figure 2F). Besides *PTPN1*, the DEGs involved in these biological functions are *ARF6, CREM*, and *FBW7*, exclusively up-regulated in the RZ, and *NAA40, HRH1, NR0B2, PFKFB2, PPR1R3B/C, PXLP1, MACROH2A1*, and *LPIN1*, down-regulated in the RZ as well as in other heart regions (Supplementary Table 4). Some of these genes, *CREM, PTPN1, HRH1* in addition to *PRCP, HSPB8*, and *STARD13* appeared to be involved in hypertrophy, pressure of the LV, altered heart ventricle contractility, and vascular endothelial cell function, representing the main dysregulated cardiovascular disease related functions found in RZ (Figure 2E, lower panel).

Overall, the gene expression analysis revealed perturbations in insulin signaling, adenosine salvage, and angiogenesis processes in the BZ whereas in the RZ, insulin signaling, carbohydrate metabolism, lipid synthesis showed to be compromised and vasoconstriction increased.

### Metabolite Profiling of Non-infarcted Left Ventricle Tissue Areas

In order to explore if the metabolic derangements observed at gene expression level, in non-infarcted areas, translate into differences in the metabolome, we performed an unbiased non-targeted LC- and GC-MS analysis to measure the metabolite levels within these areas. We were able to identify 221 metabolites putatively annotated and we applied OPLS-DA modeling to identify distinguishing metabolite levels between the BZ and RZ and healthy control LV tissue.

**Figure 3:**
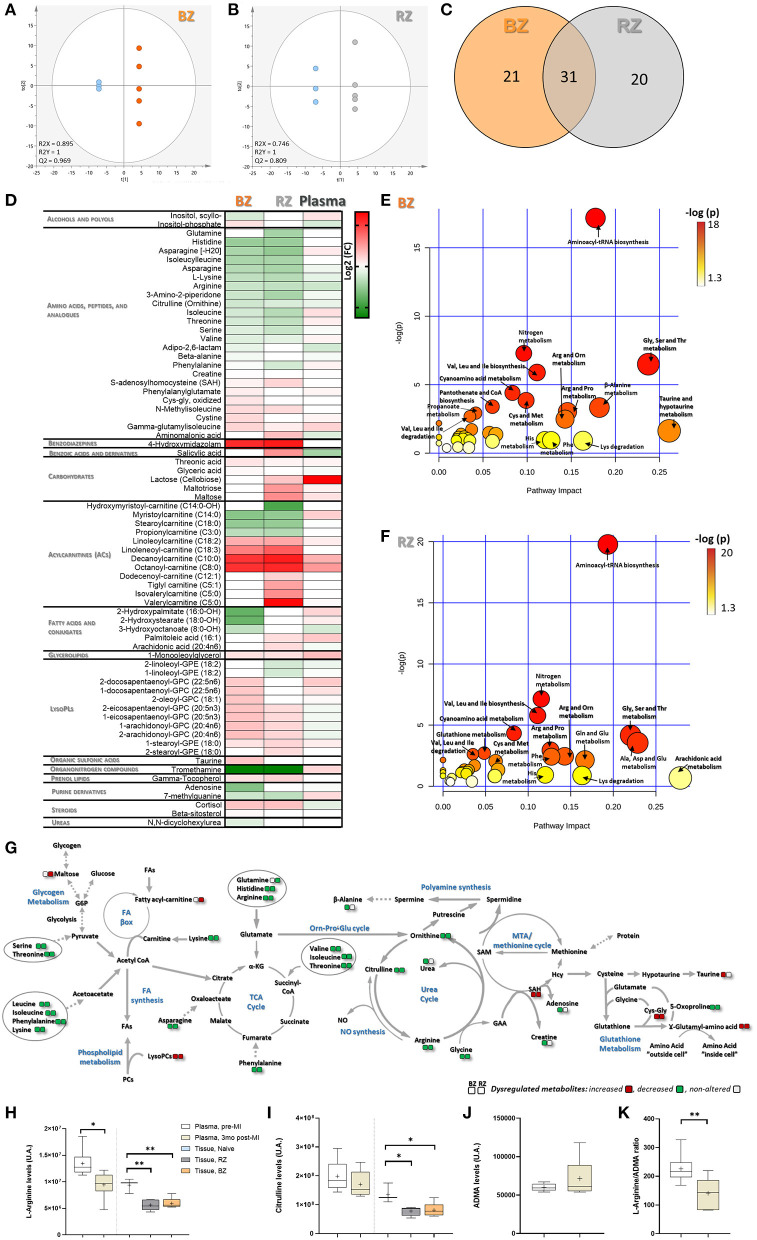
Tissue-level metabolomic characterization of remote and border zones of infarcted pig hearts. Score plot of the OPLS-DA model for: **(A)** BZ (orange) and control, LV Naïve tissue (blue) and **(B)** RZ (gray) vs. control (blue), showing clearly separation between RZ/BZ and the control group. R2 and Q2 parameters indicate a relatively good predictive capability of the models. Metabolites with VIP values >1.2 were considered as stronger contributors to discrimination among groups and included in the pathway analysis. **(C)** Venn diagram showing the overlapping differential metabolites identified from the OPLS-DA, in BZ and RZ. **(D)** Heatmap illustrating the fold change differences in metabolite levels in BZ and RZ comparing with control Naïve tissue and for plasma metabolite levels, 3 months post-MI, comparing with baseline (before MI). Red means higher abundance and green lower abundance. Refer to Supplementary Table 5 for listed fold change and *t*-test *P*-value. Pathway Analysis performed using the MetaboAnalyst tool, and MetPa for the differential metabolites in BZ **(E)** and RZ **(F)** vs. control. The color gradient is based on Fishers' exact test *P*-value (darker colors indicate more significant changes of metabolites in the corresponding pathway), whereas the circle size corresponds to the pathway impact score (the larger the circle the higher the impact score). **(G)** Schematic diagram of the perturbed metabolic pathways observed in the BZ and RZ. IZ/BZ/RZ: *n* = 5; control *n* = 3. Levels of Arginine **(H)**, Citrulline **(I)**, and ADMA **(J)** in plasma (pre- and 3 months post-MI), BZ and RZ. **(K)** Ratio Arginine/ADMA in plasma. *P*-value from unpaired (tissue) and paired (plasma) *t*-test. Plasma/BZ/RZ: *n* = 5; control *n* = 3. ***P-value: 0.0001 to 0.001; **P-value: 0.001 to 0.01; *P-value: 0.01 to 0.05.

The OPLS-DA models showed a satisfied discriminating performance between non-infarcted areas and healthy controls (Figures 3A,B). The metabolites with the highest sum of variance between groups (VIP scores >1.2), were considered potential metabolites relevant for discrimination between groups, and these were used for subsequent pathway analysis. A total of 52 and 51 metabolites met this selection criteria, in BZ and RZ, respectively (Supplementary Table 5). From these differential metabolites identified from the OPLS-DA, 31 changed in both BZ and RZ, and a total of 20 and 19 metabolites changed exclusively in the BZ and RZ, respectively (Figure 3C). In both non-infarcted areas, the metabolites contributing to the greatest variance between the groups were amino acids and derivatives, lysophospholipids (lysoPL) species and acylcarnitines (ACs) reflecting a similar metabolome profile in both zones (Figure 3D and Supplementary Figure 5).

In both tissue areas the levels of several amino acids central to energy metabolism regulation, such as histidine, lysine, arginine, citrulline, ornithine, were significantly diminished whereas in the plasma amino acid levels do not change so markedly (Figure 3D and Supplementary Table 5). Interestingly, glutamine levels seem to be reduced in the RZ (Figure 3D), suggesting dysregulation of the glutamine metabolism in this area, which strengthens the gene expression findings (Figure 2D). Depletion of all these amino acids may likely contribute to impaired TCA cycle maintenance, nitric oxide (NO) production, ornithine-proline-glutamate, and urea cycle (Figure 3G). Of note, the levels of S-adenosylhomocysteine (SAH, precursor of homocysteine, and adenosine) and γ-glutamylisoleucine (involved in the regeneration of glutathione) followed an opposite trend, increasing in both the non-infarcted tissue areas (Figure 3D), suggesting alterations in the redox balance and glutathione synthesis (Figure 3G). Despite the observed increased levels of SAH, adenosine levels appeared to be reduced in the BZ (Figure 3D).

Our results suggest that the levels of BCAAs as well as their derivative, isoleucylleucine, were diminished in both BZ and RZ, and that the levels of γ-glutamylisoleucine, involved in intracellular transport of isoleucine, and of C5 acyl carnitines generated by isoleucine and leucine catabolism, were increased in both groups (Figure 3D). These observations suggest that some BCAA catabolism is still taking place at this stage post-MI in the non-infarcted areas. Interestingly at systemic level, valine, and isoleucine show a trend of increased levels when compared with baseline (before MI) potentially indicating alterations in protein synthesis/breakdown at skeletal muscle level.

Metabolome profiling also revealed that the levels of fatty acids (FA) remain largely unchanged except for that of arachidonic acid (AA) and palmitoleic acid, which seem to be increased in the RZ (Figure 3D). Nonetheless, the ACs increased considerably mainly in the RZ (Figure 3D). In addition, the levels of lysoPL species, followed a trend of increase in both, BZ and RZ (Supplementary Figure 5), which may indicate increased turnover and/or degradation of cellular membranes and reflect higher levels of oxidative stress, since oxidative stress induces phospholipase activity. Interestingly, the data indicates a trend of higher levels of lysoPLs compared to control in the BZ than in the RZ (Supplementary Figure 6), which could reflect a higher oxidative stress in the BZ, as expected due to its proximity to the infarcted area. Also, BZ showed: (i) increased levels of oxidized amino acid forms (e.g. Cys-Glys and cysteine; Figure 3D), (ii) lower levels of the anti-oxidant β-alanine; and (iii) lower levels of γ-tocopherol, which possesses anti-inflammatory and anti-oxidative properties, in comparison to RZ (Figure 3D). Taken together these results point toward a trend of increased susceptibility to oxidative stress and reduced antioxidant capacity in the BZ compared to the RZ.

Of note is that the levels of the glycogen breakdown metabolites, including maltotriose and maltose, appeared to be increased in the RZ (Figure 3D), which corroborates gene expression data, indicating reduced carbohydrate/polysaccharide synthesis in the RZ (Figure 2F).

Pathway analysis for the differential metabolites identified in both tissue areas revealed that the most significantly dysregulated pathways were amino acid metabolism related pathways including aminoacyl-tRNA biosynthesis; nitrogen metabolism; glycine, serine, threonine metabolism; and arginine, proline, and ornithine metabolism (Figures 3E,F).

To determine if these metabolite changes could be detected at plasma level, we evaluated if the levels of the metabolites found significantly altered in the tissue were also significantly dysregulated in the plasma, three months post-MI comparing to baseline (before MI). Only arginine was found significantly dysregulated in the plasma. The levels of arginine significantly decreased at chronic phase post-MI compared with pre-MI (Figure 3H). Interestingly, related metabolites [citrulline and asymmetric dimethyl arginine (ADMA)] showed a trend of increase and decrease, respectively, although not significant (Figures 3I,J). The ratio of Arginine/ADMA in plasma three months post-MI was found significantly decreased (Figure 3K). Since ADMA inhibits NO production by competing with arginine for nitric oxide synthase (NOS) binding, a decreased ratio of Arginine/ADMA may suggest reduced NO bioavailability which in turn may induce alterations in endothelial function (20).

Overall, post-MI cardiac metabolome, in both BZ and RZ, was characterized by altered amino acid- (particularly arginine metabolism) and phospholipid metabolism. In addition, altered metabolome provided further evidence of increased exposure to oxidative stress in the BZ than in the RZ, but revealed more pronounced alterations in lipid and carbohydrate metabolism in the RZ.

### Identification of Targets Putatively Involved in Metabolism Dysregulation in Non-infarcted Left Ventricle at Chronic HF Phase

**Figure 4:**
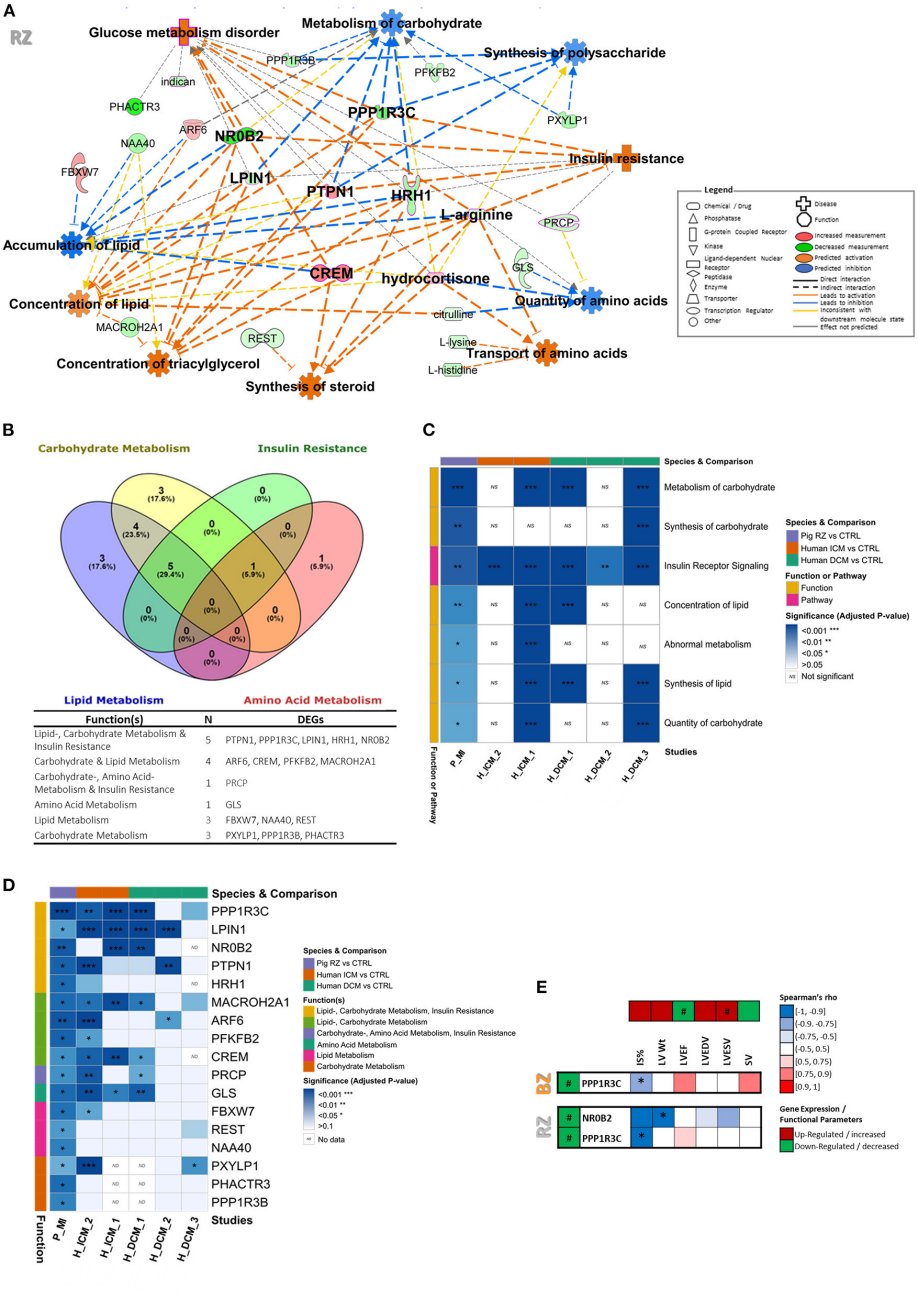
Identification of targets putatively involved in metabolic dysregulation in chronic HF and validation using datasets from human ICM and DCM patients. **(A)** Metabolic function network analysis performed in IPA for RZ using both transcriptomic and metabolomic datasets. Only the significantly dysregulated genes (FC ≥|1.5| and FDR <0.05) and metabolites (VIP >1.2 and *t*-test *p*-value <0.05) were included in the analysis. The gene or metabolite is referred to as nodes and the intensity of the node color indicates the degree of up- (red) or down- (green) regulation. *Z*-score was used to predict activation or inhibition of functions/diseases based on relationships with dataset genes and direction of change of dataset genes. **(B)** Venn diagram distributing the RZ DEGs involved in metabolic functions per metabolic category. Full metabolic function annotations per gene can be found in Supplementary Table 6. **(C)** Heatmap of enriched metabolic functions found dysregulated in RZ vs. control in the post-MI pig dataset and in GEO studies from ICM and DCM patients. More details about the GEO datasets can be found in Supplementary Table 1. **(D)** Heatmap illustrating genes involved in metabolic functions, found significantly dysregulated in both pig post-MI and human ICM/DCM datasets. For comparison of the expression levels between datasets please see Supplementary Table 7. **(E)** Heatmap illustrating Spearman's rank correlation analysis between gene expression levels and functional cardiac parameters in BZ and RZ. Only DEGs involved in metabolic function were included in this analysis. Two-tailed *P*-value of the Spearman's correlation coefficient is shown as: *P < 0.05. ^#^Demarks statistically significantly expressed genes (FDR <0.05).

To thoroughly investigate the interactions between the significantly dysregulated genes and metabolites screened out above, we performed an integrated analysis by including both omics data sets to infer a metabolic related disturbed functional interaction network and identify the key mediators involved. We focused on the RZ for this analysis, since this zone showed a more demarked disturbance of metabolic pathways and functions compared to the BZ. In line with the previously described findings, functional network analysis pointed toward activated glucose metabolism disorder, insulin resistance, synthesis of steroid; inhibited metabolism of carbohydrate, synthesis of polysaccharide, and quantity of amino acids; and altered concentration/accumulation of lipids (Figure 4A and Supplementary Table 6). The main effectors (genes/metabolites showing at least three consistent interactions leading to activation or inhibition of functions) encountered in this metabolic related functional network were: *PPP1R3C, PTPN1, NR0B2, CREM, HRH1, LPIN1*, cortisol, and arginine.

### Relevance of the Identified Targets in Human Cardiovascular Disease

To further validate the relevance of these findings in the context of human HF disease, we have compared our dataset with RNA sequencing data from LV biopsies of patients with ischemic (ICM) and dilated cardiomyopathy (DCM) (Supplementary Figure 7 and Supplementary Table 6). Specifically, we investigated if the genes involved in insulin resistance, carbohydrate-, lipid-, and amino acid metabolism functions, found dysregulated in RZ (Figure 4B) and identified as mediators in the metabolic related functional network (Figure 4A), were also significantly dysregulated in ICM and DCM patients. We used data publicly available in GEO and selected datasets that also showed significant dysregulation of some of the metabolic functions found dysregulated in our study (Figure 4C). Through this comparison, we could perceive, that 12 out of the 17 DEGs involved in metabolism dysregulation in the RZ, are also differentially expressed in the myocardium of ICM and/or DCM patients (Figure 4D). All of those 12 genes, were found dysregulated in the myocardial tissue of ICM patients, showing a high translatability of our model to human ICM disease. It is interesting to note that despite the pathophysiological differences of our chronic post-MI pig model and DCM disease we could observe some interesting overlap of dysregulated metabolic genes (10 out of the 17 DEGs involved in metabolism dysregulation in the RZ were also found dysregulated in at least one of the DCM datasets). This may suggest that the metabolic targets identified in this study in the context of chronic post-MI HF may also be involved in the pathophysiology of other HF disease-driving mechanisms such as dilated cardiomyopathy. *LPIN1* was dysregulated in four out of the five investigated human studies, followed by *PPP1R3C, MACROH2A1, GLS*, and *CREM* that were dysregulated in three of the five human studies explored.

### Correlation Between Target Gene Expression Levels and Functional Status

Lastly, to evaluate if alterations of the expression of these metabolic related genes were associated with functional consequences, a Spearman's rank correlation analysis was conducted between the gene expression levels and the parameters reflecting functional status (Figure 4E). Interestingly, both, *NR0B2* and *PPP1R3C*, showed direct significant correlations with the functionality readouts measured. Specifically, *NR0B2* levels in RZ exhibited a significantly negative correlation with LV weight and a trend of negative correlation with IS, LVESV, and LVEDV (Figure 4E). In addition, *PPP1R3C* levels in both RZ and BZ, showed a negative correlation with IS and a trend of positive correlation with LVEF (Figure 4E). *NR0B2* and *PPP1R3C* may therefore constitute important markers to elucidate pathophysiology of HF in the non-infarcted LV areas.

## Discussion

**Figure 5:**
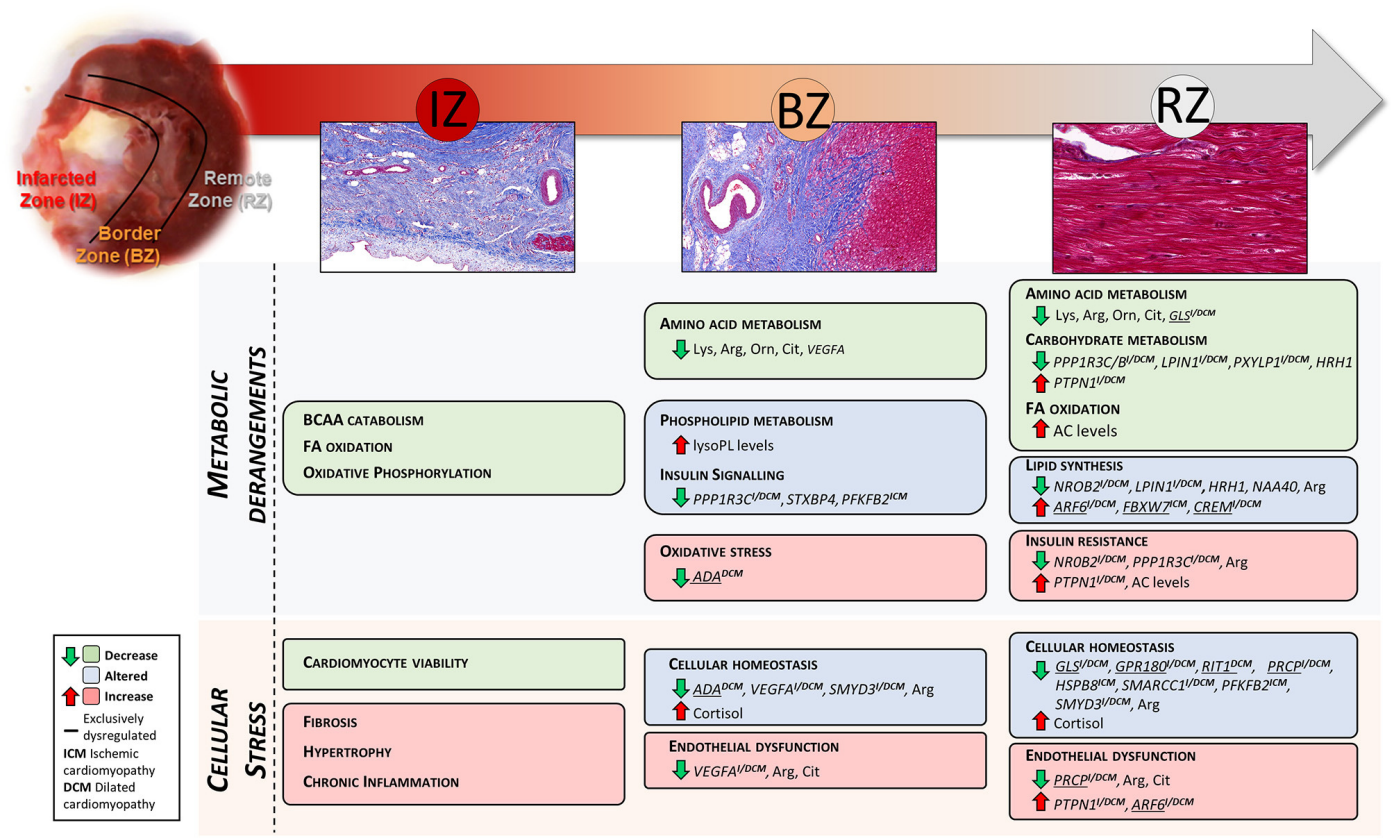
Summary of the LV region-specific metabolic and cellular derangements found in this study. Underlined genes were found significantly dysregulated exclusively in the referred tissue zone. Genes demarked with ICM and DCM were also found significantly dysregulated in LV biopsies from ischemic and dilated cardiomyopathy patients, respectively.

The present study was designed to investigate the molecular changes that occur in different LV regions of pig hearts at late stage post-MI. We focused particularly on the healthy remote area from MI, aiming to capture early dysregulated genes/metabolites potentially triggering the transition from chronic compensated to decompensated HF. Results from this work have improved the understanding of the remodeling process occurring in the non-infarcted regions of the LV and identified possible targets of further inquiry to prevent or delay HF progression, as summarized in Figure 5. The strength and novelty of our study lies on its integrative transcriptomics and metabolomics analysis approach, providing a comprehensive systemic view of the altered gene and metabolite signatures and the interconnected dysregulated pathways in chronic HF at tissue level. In addition, we have validated the transcriptomics results from our post-MI pig model with data from ICM and DCM patient biopsies, which confirmed the translatability of our model and strengthened the relevance of the findings reported in the context of human HF disease.

This study shows a coordinated dysregulation of metabolic related pathways in the infarcted and non-infarcted areas of the LV (Figure 5). Due to the replacement of the cardiac muscle by scar tissue in the infarcted area, oxidative metabolism and BCAA catabolism were reduced. Whereas, non-infarcted areas showed altered amino acid-, carbohydrate-, and lipid metabolism and perturbed insulin signaling.

The cardiac metabolome in the non-infarcted areas revealed a significant depletion of amino acids. This might be due to increased heart's reliance on amino acids due to a high myocardium anabolic activity and/or to increased protein synthesis due to cardiac hypertrophy. Nonetheless, no macroscopic regional ventricular hypertrophy was observed in this model. Compensatory hypertrophy at cellular level is expected to occur in the remote area after MI, however no signatures of cardiac hypertrophy were detected at gene level {i.e., activation of fetal gene program, decreased expression of FAO enzymes in favor of glycolytic ones, up-regulation of signaling pathways involved in hypertrophy [as PI3K, AKT, AMP-activated protein kinase, and mTOR (21)]}. Also circulating amino acids do not follow exactly the same trend as in the heart, suggesting that the observed changes may also reflect alterations in organism level regulatory pathways, such as skeletal muscle metabolism. Further studies should be performed to investigate whether the observed depletion of amino acids and other energy substrates in the non-infarcted area is owing to increased consumption, decreased production, or both. Amino acid depletion characteristically reflects cardiac metabolic stress events such as impaired TCA cycle, inadequate oxidative phosphorylation, ATP depletion, impaired protein synthesis, and increased oxidative stress (22). There are still unknowns on the role of amino acid metabolism in the progression of HF, however a previous study showed that chronic HF patients have reduced arterial amino acids levels, and that progressive reductions in arterial amino acid levels were associated with progressive LV deterioration (23).

Specifically, our data showed that the levels of lysine, arginine, and citrulline/ornithine were significantly reduced in both BZ and RZ, compared to the healthy LV controls. Lysine is required to produce both, collagen and carnitine. Collagen is crucial to strength the artery walls, and carnitine is essential for the transport of FAs through the mitochondria for oxidation, thus it would be interesting to further explore the impact of altered lysine levels in the progression of HF. In addition, the significant decrease in arginine and citrulline/ornithine levels suggests perturbations in NO metabolism, urea cycle, and polyamine synthesis, which previously has been linked to endothelial dysfunction, pro-inflammatory signaling and development of cardiac hypertrophy (24). Of note, arginine levels, as well as the ratio arginine/ADMA, were found significantly diminished in the plasma, potentially suggesting reduced NO bioavailability and consequently alterations in vascular function. A low arginine/ADMA ratio has been associated with high risk of cardiovascular disease events (25). Supplementation with L-arginine may be able to restore the physiological status by normalizing the extracellular arginine to ADMA ratio.

Interestingly, the decreased levels of glutamine and *GLS* expression in the RZ suggest a defective glutamine degradation metabolism in this LV region. In agreement, ICM and DCM patients also showed significantly lower expression of *GLS* in LV compared with controls (Supplementary Figure 7). Glutamine degradation promotes cardiovascular metabolism homeostasis contributing to maintain cellular amino acid levels, to support anaplerosis of TCA cycle constituents and to stimulate antioxidant defense response through glutathione production (26). Liu et al., demonstrated that glutamine metabolism in rat hearts is depressed during ischemia/reperfusion injury and that inhibition of miR-200c, which directly targets *GLS*, is cardioprotective (27). Moreover, some clinical trials have shown beneficial effects of glutamine supplementation in patients with coronary heart disease (28), and a prospective study revealed that dietary intake of glutamine is inversely related to the risk of cardiovascular mortality (29). In view of our and previous results, amelioration of glutamine metabolism, by either glutamine replenishment and/or therapeutic targeting of *GLS*, might have a potential to serve as a strategy for preventing cardiac dysfunction, particularly in the RZ of the LV.

In addition to the substantial alterations in the amino acid metabolism, our data revealed dysregulation of lipid and carbohydrate metabolism in the non-infarcted areas, particularly in the RZ. These findings were also manifested by increased tissue levels of ACs and lysoPLs, and dysregulation of genes enriched in carbohydrate and lipid metabolism related pathways/functions.

It is well-known that ACs accumulate in states of impaired FAO, which could result from defects in mitochondrial FAO enzymes or increased FAO relative to TCA flux. Since no changes in the expression of FAO enzymes were observed, the second option is most likely the cause. The rates of FA uptake and oxidation are probably increased in the RZ to compensate for the deficiency in energy production in the IZ. This in turn, may produce a “bottleneck” of substrate flux into the TCA cycle that eventually leads to the accumulation of FAO intermediates, namely ACs. In fact, several studies have shown that myocardial decompensation during HF is associated with extensive changes in lipid metabolism, including impaired FAO and accumulation of ACs (30). Ahmad et al. reported that plasma LCAC C18:2 was significantly higher in patients with end-stage HF (30). In agreement, our data showed that, C18:2, was significantly up-regulated in both BZ and RZ. Considering all the adverse roles attributed to high levels of LCACs [i.e., proinflammatory, arrhythmogenic, ROS production, and insulin resistance (31, 32)], our data suggests that it could be beneficial to decrease LCAC production, in the non-infarcted areas, by for example promoting increased glucose oxidation, decreasing FAO flux, or enhancing TCA cycle flux by amino acid replenish therapy.

Another interesting finding of this study is the perturbed insulin signaling, predominantly in the RZ. In addition to affect substrate selection, insulin can influence mitochondrial biogenesis and function and it is likely that altered insulin signaling may precede maladaptive metabolic remodeling in the non-infarcted area. Altered gene and metabolite signatures linked to insulin resistance encountered in the RZ include: (i) accumulation of LCACs; (ii) decreased polysaccharide synthesis as evidenced by accumulation of glycogen metabolites (e.g., maltotriose, maltotetraose, and maltopentaose) and decreased expression of *PPP1R3B*/*C*; (iii) lower levels of arginine; and (iv) increased expression of *PTPN1*.

At gene level, we can highlight, *CREM, NR0B2, LPIN1, PPP1R3C*, and *PTPN1*, identified as main effectors in a metabolic function network analysis and found dysregulated also in ICM and/or DCM patients.

*CREM* is involved in lipid metabolism homeostasis and was exclusively up-regulated in RZ. Different mouse models with gain or loss of CREM function suggested CREM as a key regulator of gene expression in the pathogenesis of HF, being involved in the regulation of genes encoding mitochondrial enzymes and cardiac contractile proteins. CREM inactivation has been proven to protect cardiomyocytes from hypertrophy, fibrosis, and contribute to improve LV function (33). However, the impact of CREM and its direct targets on maladaptive remodeling of the remote non-infarcted area at late stage after MI warrants further validation.

*NR0B2* (or *SHP*), also involved in lipid metabolism, was down-regulated in all LV areas, but more pronouncedly in the RZ, and *NR0B2* levels in the RZ followed a significant inverse correlation with LV weight and a trend of correlation with other functional measurements. A previous study reported that SHP-deficient mice show significantly higher LV masses than WT mice and that SHP blocked the development of hypertrophy in cardiomyocytes (34). Conversely, SHP upregulation upon high-fat feeding was shown to lead to lipid accumulation, insulin resistance, and inflammation in cardiomyocytes (35). Therefore, further follow-up studies are needed to better understand whether changes in *NR0B2* during HF progression are compensatory or maladaptive.

*LPIN1*, down-regulated in IZ and RZ, encodes lipin 1 protein, which acts as a nuclear transcriptional coactivator for PPARGC1A/PPARA, modulating FAO gene expression. It also participates in glycerolipid metabolism and triglyceride synthesis. *LPIN1* is highly expressed in myocardium, is induced by a physiologic stimulus (β-adrenergic agonism) to increase FAO gene expression, and downregulated by a pathophysiologic stimulus (heart failure or hypertrophy) (36). Evidence has emerged that lipin-1 deficiency leads to cardiac dysfunction in mice due to lipodystrophy, modified hormonal regulation, and fuel availability (37). Interestingly, from the metabolic genes in analysis, *LPIN1* was the one found dysregulated in more human ICM/DCM studies, which highly suggests that *LPIN1* possesses a key role in HF pathophysiology.

*PPP1R3C* and other members (*PPP1R3A* and *PPP1R3B*) of the PP1-GTS (protein phosphatase-1 -glycogen targeting subunit) gene family were found down-regulated in the different LV areas. Notably, we identified a significant direct inverse correlation between *PPP1R3C* expression levels and IS in both BZ and RZ, suggesting that this glycogen-associated regulatory subunit might be of importance in the pathophysiology of HF in the non-infarcted LV. Contrary to *PPP1R3A*, previously described as a central regulator in HF pathology (38), the specific role of *PPP1R3C* in HF pathology has not been investigated yet. Nevertheless, previous findings demonstrated that mice with a heterozygous deletion of *PPP1R3C* have reduced glycogen stores in the heart and develop progressive glucose intolerance and insulin resistance with aging (39).

*PTPN1* is of particular interest, since it encodes PTP1B, a negative regulator of the insulin receptor signaling and the vascular endothelial growth factor A (VEGFA), contributing therefore to both insulin resistance and endothelial dysfunction. *PTPN1* expression is significantly increased in LV samples of patients with systolic dysfunction (40), and previous studies showed that pharmacological inhibition of PTP1B improves glucose homeostasis, insulin signaling, cardiac VEGF signaling, angiogenesis, and protects against chronic afterload-induced HF in mice (41). In addition, PTP1B is described as a regulator of the unfolded protein response, playing a major role in the development of ER stress, via regulation of PRKR-like Endoplasmic Reticulum Kinase (PERK) and Inositol-Requiring Enzyme 1 alpha (IRE1α). PTP1B has shown to dephosphorylate/inactivate PERK and activate IRE1α leading to restoration of bulk protein synthesis in several tissues and cell lines (42, 43). This may suggest a connection between the increased expression of PTP1B and the decreased amino acid levels, potentially due to increased protein synthesis, observed at metabolic level in the non-infarcted area. Characterization of the fluctuations of PTP1B and its direct targets at protein and phosphoprotein level, in the non-infarcted area, would provide further insights into the role of PTP1B in maladaptive remodeling at chronic MI stage.

Our data also point toward increased oxidative stress particularly in the BZ, and decreased cellular homeostasis and altered endothelial function in both BZ and RZ areas. Specifically, a simultaneous significant decrease in arginine and citrulline levels in both BZ and RZ, arginine and arginine/ADMA in the plasma, and altered expression of *VEGFA* and *ADA* in BZ, and *PRCP, PTPN1*, and *ARF6* in the RZ, strongly suggests reduced NO bioavailability and vascular remodeling in the non-infarcted area. It has been reported that *ADA* expression and enzyme activity in the heart is decreased in chronic HF patients with increasing pathologic signs of the disease (44), as a compensatory mechanism to reduce degradation of adenosine. Likewise, *ADA* was significantly down-regulated in the BZ where the levels of adenosine were diminished, reflecting an adaptative response to enhance intracellular adenosine levels and consequently its cardioprotective role. Notably, *ADA* was also found reduced in DCM patients (Supplementary Table 7), providing further evidence that *ADA* might be an important marker in the management of HF.

## Study Limitations

There are some limitations in this study that need to be considered. This study was performed with tissue samples from a pig HF model so extrapolations to humans should be carefully evaluated.

Another, and probably the major, limitation of this study is the relatively small sample size in each group, which might prevent some of the differences, specifically in the levels of metabolites, from being fully apparent. Also, despite that we have performed an untargeted metabolomic analysis, we were not able to find an annotated ID for all the putative hits detected (221 over a total of 1,674 hits) which limited the breadth of the analysis. Nevertheless, the identified metabolites are involved in key cellular metabolism pathways, namely on lipid, carbohydrate, amino acid, and urea metabolism.

Additionally, some findings reported herein were not entirely consistent with previously reported results in HF patients, such as decreased levels of 7-methylguanine and increased levels of BCAA. This may be due to: (i) different model characteristics, including the stage and severity of the HF disease at the time of analysis, (ii) differences in the myocardial vs. peripheral metabolism since the majority of the metabolomic results reported in the literature were performed on plasma, and (iii) region-specific variances between infarcted and non-infarcted LV areas.

Finally, this study should be considered hypothesis-generating and future studies are needed to validate our findings in larger chronic HF and no-HF cohorts. Gene expression validation *in vitro* and *in vivo* in HF animal models (including gene overexpression and/or knockdown) should be carried out and changes at protein level as well as associated post-translational modifications should be investigated to elucidate the mechanisms by which the identified candidate targets reflect, generate, and/or exacerbate HF pathophysiology.

## Conclusion

In summary, the present study suggests that insulin signaling, amino acid-, lipid-, and carbohydrate metabolism and endothelial function are primarily altered in non-infarcted areas in chronic HF. Altogether, our findings strongly suggest that a combined therapeutic strategy capable to prevent both cardiomyocyte metabolism dysregulation and endothelial dysfunction could be an attractive approach to HF treatment. The repository of genes and metabolites identified as dysregulated in the healthy non-infarcted LV region may precede and/or accompany progressive transition toward maladaptive remodeling and cardiac dysfunction and their potential value as therapeutic targets to counteract the pathophysiology of HF would be of interest to investigate further.

## Data Availability Statement

The RNA sequencing data can be accessed at ArrayExpress (https://www.ebi.ac.uk/arrayexpress/experiments/E-MTAB-8856/).

## Ethics Statement

All animal procedures were undertaken according to the guidelines from Directive 2010/63/EU of the European Parliament on the protection of animals used for scientific purposes. The study was approved by the local Ethical Committee in Gothenburg, Sweden, 2016-06-29 (permit no 68 2016). Written informed consent was obtained from the owners for the participation of their animals in this study.

## Author Contributions

CC, Q-DW, PS, KJ, and JS: conceptualization. CC, Q-DW, LC, JW, KJ, and JS: methodology. CC, AW, GL, MB, and KR-M: investigation. CC, AW, and BU: formal analysis. CC: writing—original draft. CC, Q-DW, LC, BU, JW, PS, KJ, and JS: writing—review and editing. PS and JS: funding acquisition. Q-DW, KJ, and JS: resources and supervision. All authors contributed to the article and approved the submitted version.

## Funding

This work was supported by AstraZeneca and the University of Skövde, Sweden under grants from the Knowledge Foundation [2014/301, 2016/294, and 2016/330]. The funder was not involved in the study design, collection, analysis, interpretation of data, the writing of this article, or the decision to submit it for publication.

## Conflict of Interest

CC, Q-DW, GL, LC, AW, KR-M, MB, JW, and PS are employed by AstraZeneca. The remaining authors declare that the research was conducted in the absence of any commercial or financial relationships that could be construed as a potential conflict of interest.

## Publisher's Note

All claims expressed in this article are solely those of the authors and do not necessarily represent those of their affiliated organizations, or those of the publisher, the editors and the reviewers. Any product that may be evaluated in this article, or claim that may be made by its manufacturer, is not guaranteed or endorsed by the publisher.
