# Supplementary material for: Unraveling the Metabolic Derangements Occurring in Non-infarcted Areas of Pig Hearts With Chronic Heart Failure
Source: Front Cardiovasc Med. 2021 Oct 13;8:753470. doi: 10.3389/fcvm.2021.753470 (PMC8548620; doi:10.3389/fcvm.2021.753470)
Supplement: Supplementary file 1 [file Data_Sheet_1.docx]

**Supplementary Material**

***Unravelling the metabolic derangements occurring in non-infarcted areas of pig hearts with chronic heart failure***

# Cláudia Correia^1,2,*^, Qing-Dong Wang^2^, Gunilla Linhardt^2^, Leif Carlsson^2^, Benjamin Ulfenborg^1^, Anna Walentinsson^3^, Katarina Rydén-Markinhutha^2^, Margareta Behrendt^2^, Johannes Wikström^2^, Peter Sartipy^1,4^, Karin Jennbacken^2^, Jane Synnergren^1,*^

**Expanded Materials & Methods**

**Figures S1 - S6**

**Tables S1 – S7**

**Expanded Materials & Methods**

**Induction of myocardial infarction**

Starting three days before MI surgery, amoxicillin (280 mg, twice daily) was administered for six days. Two days before surgery antithrombotic prophylaxis with clopidogrel (75 mg) and aspirin (250 mg) once daily were initiated and continued until the day of termination. On the day of surgery, anesthesia was initiated by intramuscular administration of ketamine (10 mg/kg) and midazolam (2 mg/kg) followed by propofol (2.5-3.5 mg/kg) injected via the marginal ear vein. Subsequently, the pigs were intubated for artificial ventilation and continuous inhalation of isoflurane (2-3% mixed with room air and 10% O_2_) for the remainder of the procedure. Tidal volume and respiration rate were adjusted to maintain arterial blood gases and pH within normal physiological ranges and these were checked repeatedly during the surgery by means of a blood gas analyzer (ABL 825, Radiometer, Denmark). Additional analgesia (buprenorphine, 0.05-0.1 mg/kg), was intravenously injected before surgery. By means of the Seldinger technique, two venous catheters were placed in the vena saphena on each hind leg for blood sampling and infusion of lidocaine (2 mg/kg as a bolus and 1 mg/min infusion to mitigate ventricular arrhythmias) and dopamine (10 µg/kg/min) in case of hypotension due to ventricular tachycardia. ECG-electrodes to record ECG (lead I, II, III, V1 and V2) as well as patches were placed on the chest to enable direct-current cardioversion (at 150 - 200 J) in case of ventricular fibrillation. Continuous fluid replacement (Rehydrex, 5 mL/kg/h) and heparin (250-300 IE/kg) were given intravenously. A catheter (Seldinger introducers) was placed in the right carotid artery for catheterization of the LAD. In the carotid artery introducer, a guide (GC Adroit, 6F, JR4, Johnson & Johnson, Sweden) were advanced to the aortic arch and by means of fluoroscopic guidance and injection of contrast agent (Visipaque 320, Apoteket, Sweden), the coronary vessels were visualized to enable positioning of a guide wire (PCI Wire, Wizdom SGW 0.014’’ 300 J Soft, Johnson & Johnson, Sweden or Choice Floppy guide wire H74) in the LAD.

Post-surgery analgesia (flunixin, 2.2 mg/kg) was given intramuscularly for maximum three days post-MI surgery.

Three months after the MI procedure the pigs were sacrificed. Hence, the pigs were anesthetized as previously described and given an additional intravenous bolus dose (20-30 mg) of propofol. Subsequently, the isoflurane inhalation was increased to reach a minimum alveolar concentration (MAC) of 1.5 (surgical anaesthesia depth) before the chest was opened and the heart excised and washed in chilled saline and chilled phosphate-buffered saline.

**Transcriptomics analysis**

**Pathway and functional network analysis**

GO terms and KEGG pathways with a p-value<0.05 (Benjamini-Hochberg correction) were considered significantly enriched. The significance of the associations between the dataset and canonical pathways/biological functions were measured using two different approaches: (i) a ratio of the number of genes from the dataset that map to the pathway divided by the total number of genes that map to the pathway / biological function stored in the Ingenuity Knowledge Base, and (ii) a Right-Tailed Fisher’s Exact Test that reflects the likelihood that the association or overlap between a set of significant molecules in the dataset and a given process/pathway is due to random chance. The analysis also calculates a Z-score that compares the direction of change of a molecule to what was expected from the literature. If the observed direction of change was mostly consistent with a particular activation state of the pathways/biological functions (“activated” or “inhibited”), then a prediction is made about the activation state of enriched pathways/biological functions. Both direct and indirect relationships were considered for the functional network analysis.

**Metabolomics analysis**

**Materials**

Solvents: Methanol, HPLC-grade was obtained from Fischer Scientific (Waltham, MA, USA) Chloroform, Suprasolv for GC was obtained from Merck (Darmstadt, Germany) Acetonitrile, HPLC-grade was obtained from Fischer Scientific (Waltham, MA, USA) 2-Propanol, HPLC-grade was obtained from VWR (Radnor, PA, USA) H2O, Milli-Q. Reference and tuning standards: Purine, 4 μM, Agilent Technologies (Santa Clara, CA, USA) HP-0921 (Hexakis(1H, 1H, 3H-tetrafluoropropoxy)phosphazine), 1 μM, Agilent Technologies (Santa Clara, CA, USA) Calibrant, ESI-TOF, ESI-L Low Concentration Tuning Mix, Agilent Technologies (Santa Clara, CA, USA) HP-0321 (Hexamethoxyphosphazine), 0.1 mM, Agilent Technologies (Santa Clara, CA, USA). Stable isotopes internal standards: LC-MS internal standards: 13C9-Phenylalanine, 13C3-Caffeine, D4-Cholic acid, D8-Arachidonic Acid, 13C9-Caffeic Acid were obtained from Sigma (St. Louis, MO, USA). GC-MS internal standards: L-proline-13C5, alpha-ketoglutarate-13C4, myristic acid-13C3, cholesterol-D7 were obtained from Cil (Andover, MA, USA). Succinic acid-D4, salicylic acid-D6, L-glutamic acid-13C5,15N, putrescine-D4, hexadecanoic acid-13C4, D-glucose-13C6, D-sucrose-13C12 were obtained from Sigma (St. Louis, MO, USA).

**Sample preparation**

Extraction was performed as previously described(1). Depending on the weight of the myocardial tissue samples, 800-1200 µL of extraction buffer (90/10 v/v methanol: water) including internal standards were added to the samples. The samples were shaken at 30 Hz for 2 min in a mixer mill with two tungsten beads. After centrifugation (at +4°C, 14 000 rpm, for 10 min), 200 µL (LC-MS) and 50 µL (GC-MS) of the supernatant were transferred to micro vials and solvents were evaporated to dryness.

A small aliquot of the remaining supernatants was pooled and used to create quality control (QC) samples. MSMS analysis (LC-MS) was run on the QC samples for identification purposes. The samples were analyzed in batches according to a randomized run order on both GC-MS and LC-MS.

**GC-MS Analysis**

Derivatization and GC-MS analysis were performed as described previously(1). The derivatized sample (0.5 μL) was injected in splitless mode by a L-PAL3 autosampler (CTC Analytics AG, Switzerland) into an Agilent 7890B gas chromatograph equipped with a 10 m x 0.18 mm fused silica capillary column with a chemically bonded 0.18 μm Rxi-5 Sil MS stationary phase (Restek Corporation, USA). The injector temperature was 270°C, the purge flow rate was 20 mL/min and the purge was turned on after 60 sec. The gas flow rate through the column was 1 mL/min, the column temperature was held at 70°C for 2 min, then increased by 40 °C min^-1^ to 320°C and held there for 2 min. The column effluent was introduced into the ion source of a Pegasus BT time-of-flight mass spectrometer, GC/TOFMS (Leco Corp., St Joseph, MI, USA). The transfer line and the ion source temperatures were 250°C and 200°C, respectively. Ions were generated by a 70 eV electron beam at an ionization current of 2.0 mA, and 30 spectra/sec were recorded in the mass range m/z 50-800. The acceleration voltage was turned on after a solvent delay of 150 sec. The detector voltage was 1800-2300 V.

**LC-MS Analysis**

Before LC-MS analysis the sample was re-suspended in 10 + 10 µL methanol and water. All samples were first analyzed in positive mode. Thereafter, the instrument was switched to negative mode and a second injection of each sample was performed.

The chromatographic separation was performed on an Agilent 1290 Infinity UHPLC-system (Agilent Technologies, Waldbronn, Germany). Each sample (2 uL) was injected onto an Acquity UPLC HSS T3, 2.1 x 50 mm, 1.8 μm C18 column in combination with a 2.1 mm x 5 mm, 1.8 μm VanGuard precolumn (Waters Corporation, Milford, MA, USA) held at 40°C. The gradient elution buffers were A (H2O, 0.1% formic acid) and B (75/25 acetonitrile:2-propanol, 0.1% formic acid), and the flow-rate was 0.5 mL/min. The compounds were eluted with a linear gradient consisting of 0.1-10% B over 2 min, B was increased to 99% over 5 min and held at 99% for 2 min; B was decreased to 0.1% for 0.3 min and the flow-rate was increased to 0.8mL/min for 0.5 min; these conditions were held for 0.9 min, after which the flow-rate was reduced to 0.5 mL/min for 0.1 min before the next injection.

The compounds were detected with an Agilent 6550 Q-TOF mass spectrometer equipped with a jet stream electrospray ion source operating in positive or negative ion mode. The settings were kept identical between the modes, with exception of the capillary voltage. A reference interface was connected for accurate mass measurements; the reference ions purine (4 μM) and HP-0921 (Hexakis(1H, 1H, 3H-tetrafluoropropoxy)phosphazine) (1 μM) were infused directly into the MS at a flow rate of 0.05 mL/min for internal calibration, and the monitored ions were purine m/z 121.05 and m/z 119.03632; HP-0921 m/z 922.0098 and m/z 966.000725 for positive and negative mode respectively. The gas temperature was set to 150°C, the drying gas flow to 16 L/min and the nebulizer pressure 35 psig. The sheath gas temp was set to 350°C and the sheath gas flow 11 L/min. The capillary voltage was set to 4000 V in positive ion mode, and to 4000 V in negative ion mode. The nozzle voltage was 300 V. The fragmentor voltage was 380 V, the skimmer 45 V and the OCT 1 RF Vpp 750 V. The collision energy was set to 0 V. The m/z range was 70-1700, and data was collected in centroid mode with an acquisition rate of 4 scans/sec (1977 transients/spectrum).

**Data processing and analysis**

For the GC-MS data, all non-processed MS-files from the metabolic analysis were exported from the ChromaTOF software in NetCDF format to MATLAB R2016a (Mathworks, Natick, MA, USA), where all data pre-treatment procedures, such as base-line correction, chromatogram alignment, data compression and Multivariate Curve Resolution were performed using custom scripts. The extracted mass spectra were identified by comparisons of their retention index and mass spectra with libraries of retention time indices and mass spectra(2). Mass spectra and retention index comparison was performed using NIST MS 2.0 software. Annotation of mass spectra was based on reverse and forward searches in the library. Masses and ratio between masses, indicative of a derivatized metabolite, were especially notified. If the mass spectrum according to SMC’s experience was with the highest probability indicative of a metabolite and the retention index between the sample and library for the suggested metabolite was ± 5 (usually less than 3) the deconvoluted “peak” was annotated as an identification of a metabolite. Metabolites were identified with the HMDB database (http:// hmdb.ca).

**Supplemental Figures and Figure Legends**


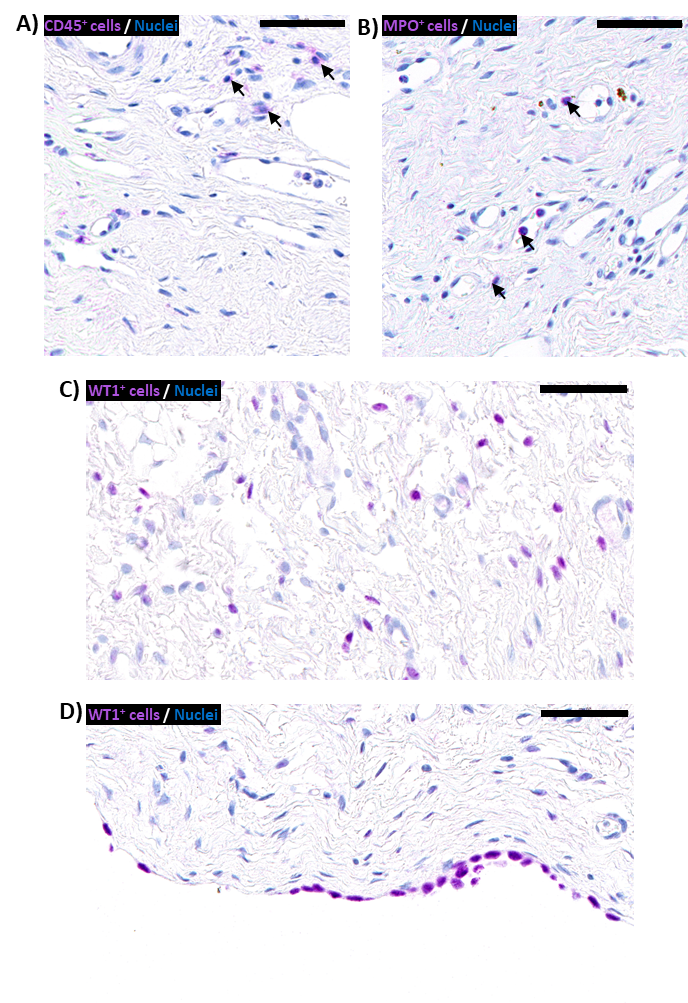


**Figure S1. Infiltration of inflammatory cells and Epicardium-Derived Cells (EPDCs) in the Infarcted Zone (IZ).** Staining for hematopoietic lineage marker CD45 **(A)**, for the neutrophil specific marker MPO **(B)** and for the EPDC specific marker WT1 **(C-D)** is shown in purple. Nuclei are stained with DAPI (blue). Scale bars: 50µm.


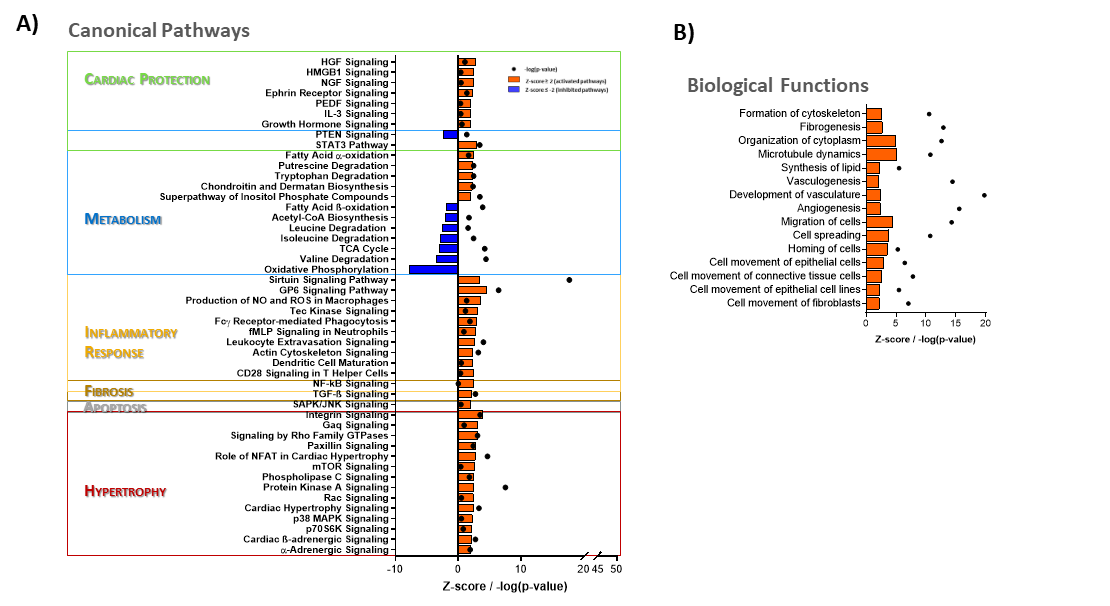


**Figure S2. Altered canonical pathways and biological functions in the Infarcted Zone (IZ).** Significantly dysregulated canonical pathways **(A)** and biological functions **(B)** were determined by p-value of overlap between pathway molecules and the DEGs in the IZ using Ingenuity pathways analysis (IPA). Blue indicates inhibited and orange indicates activated pathway/function (negative and positive overall z-scores, respectively). The threshold for differential expression was set to FC>2 with an FDR<0.05.


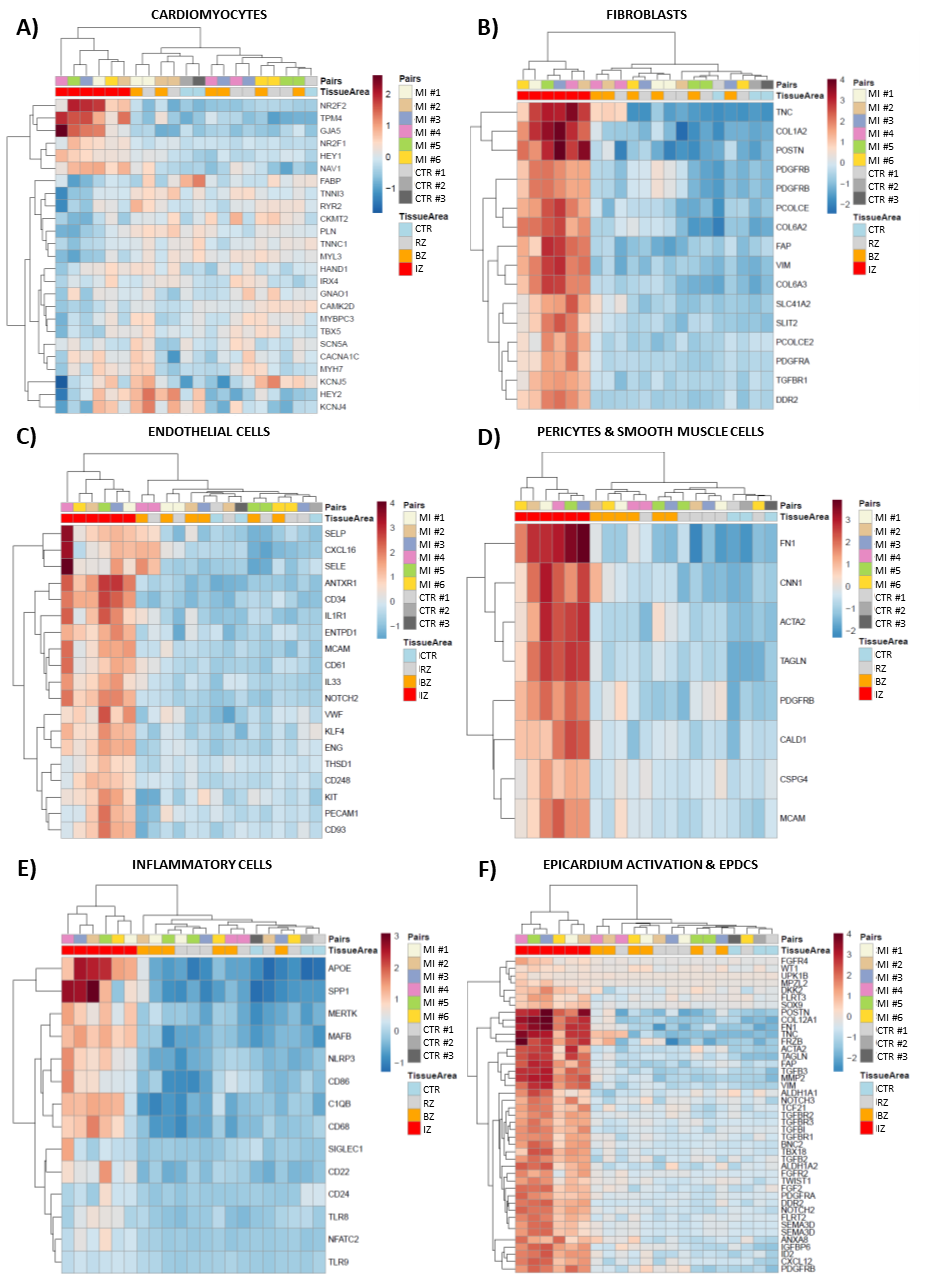


**Figure S3. Characterization of cell-type–specific gene expression in the different myocardium areas.** **A-F)** Heatmap images of gene expression profiles, illustrating a trend towards decreased expression of some cardiomyocyte markers **(A)** and increased expression of fibroblast **(B)**, endothelial **(C),** mural cells **(D)**, inflammatory cells **(E)** specific genes in the infarcted area. The infarcted area also shows a distinct up-regulation of epicardium activation and EPDC related genes **(F)**. Red indicates relatively high expression of a given gene, and blue indicates relatively low expression. Lighter shades and white represent genes with intermediate expression levels. Samples and genes have been reordered by hierarchical clustering as illustrated with the dendrogram. Heatmaps were created based on FPKM values to identify consistent changes in expression profiles between heart areas.

**
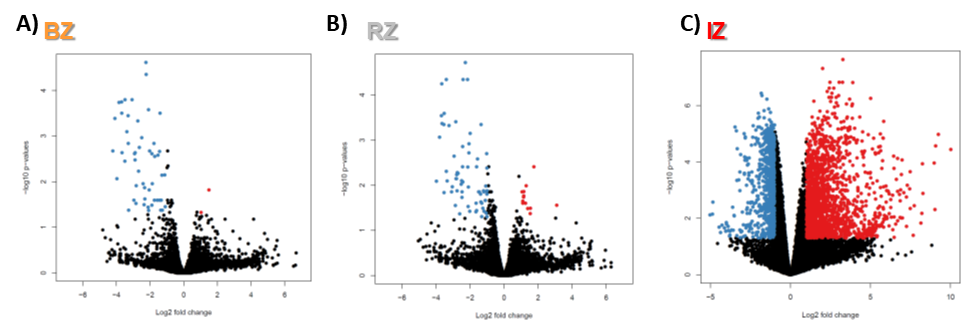
**

**Figure S4. Differentially expressed genes in the different myocardium areas.** Volcano plots displaying the log_2_ fold change vs. -log10 of the BH adjusted P-value for all genes in the border zone (BZ) **(A)**, remote zone (RZ) **(B)** and infarcted zone (IZ) **(C)** comparing with control tissue of Naïve LV. Differentially expressed genes (DEGs) are highlighted: red=up-regulated, blue=down-regulated.


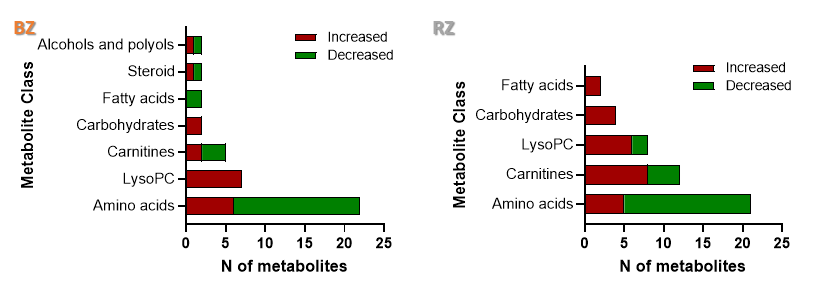


**Figure S5. Dysregulated metabolite classes for border- (BZ) and remote- (RZ) myocardial zones**. Metabolite class categories were determined using the Human Metabolome Database (HMDB).

**Figure S6. Dysregulation of amino acids and lipid metabolism in border zone (BZ) and remote zone (RZ).** Violin plots showing the levels of amino acids, LysoPL and acylcarnitines found dysregulated in BZ (orange) and RZ (grey) comparing to Naïve control LV tissue (blue). Median of the data and the interquartile range are indicated.


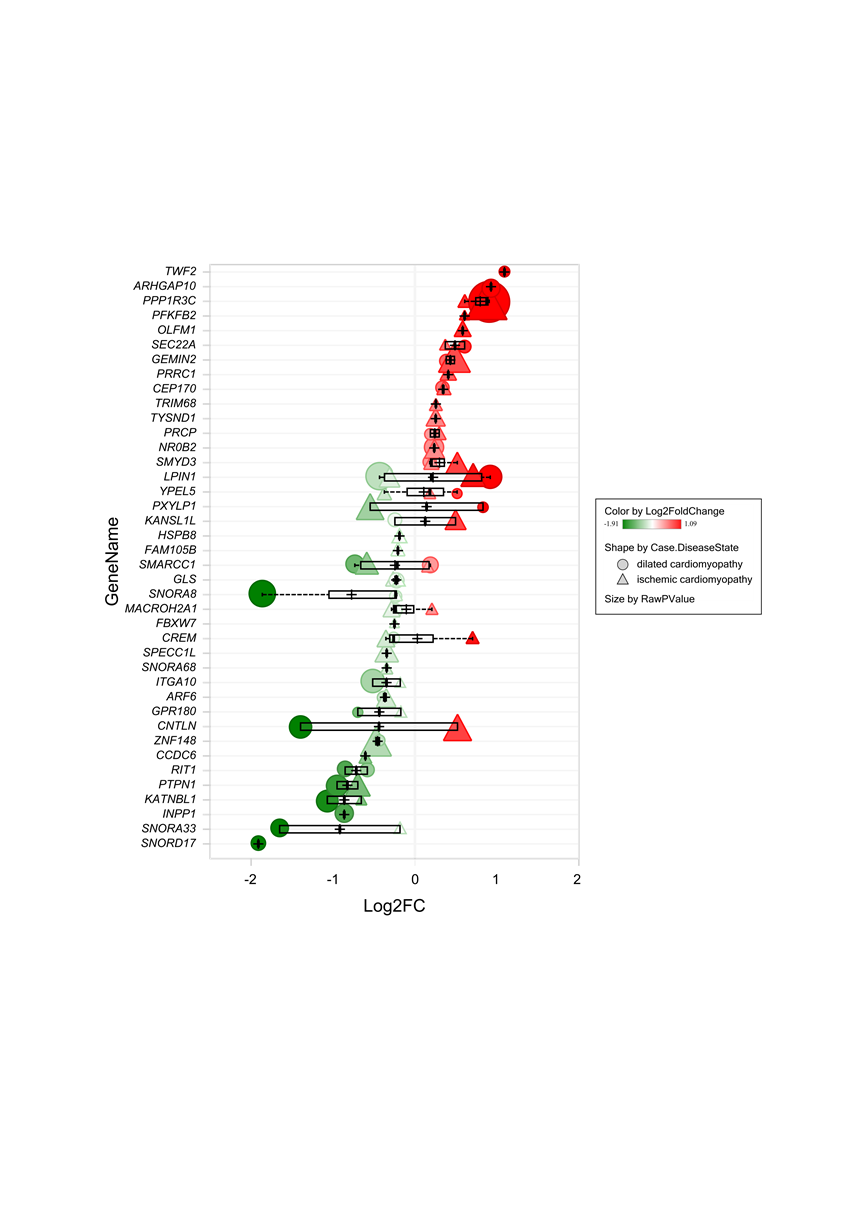


**Figure S7. RZ DEGs found significantly dysregulated in LV biopsies of patients with Ischemic and dilated cardiomyopathy.**

**Supplemental Tables**

**Table S1. Geo Datasets from human ischemic (ICM) and dilated cardiomyopathy (DCM) used for comparison with the generated Post-MI Pig dataset**

| **GEO Dataset** | **Study** | **Number of samples** | **Tissue Characteristics** | **Platform** | **Patients information** | **Reference** |
| --- | --- | --- | --- | --- | --- | --- |
| [GSE5406](https://www.ncbi.nlm.nih.gov/geo/query/acc.cgi?acc=GSE5406) | H ICM1 | n=108 ICM Patients vs n=16 nonfailing control | Left ventricular myocardium | GPL96 [HG-U133A] Affymetrix Human Genome U133A Array | - Myocardium was obtained from patients undergoing heart transplantation for advanced systolic heart failure and from nonfailing controls deemed unsuitable for transplantation. - NYHA class 3 or 4 patients with LV EF of 14±8%. Nonfailing controls had normal LV EF of 56±7% (P=0.0001 versus failing). - Ages were comparable in subjects with HF (57±12 years) and nonfailing controls (54±12; P=0.3). | (3) |
|  | H DCM1 | n=86 DCM Patients vs n=16 nonfailing control |  |  |  |  |
| [GSE1145](https://www.ncbi.nlm.nih.gov/geo/query/acc.cgi?acc=GSE1145) | H ICM2 | n=11 ICM patients vs. n=11 normal controls | Left ventricle biopsy | GPL570 [HG-U133_Plus_2] Affymetrix Human Genome U133 Plus 2.0 Array - 54,675 probes -20,283 genes. | - Samples were collected from patients undergoing cardiac transplantation. The normal control myocardial samples were collected from normal organ donors whose hearts could not be used for transplants. | [http://www.cardiogenomics.org](http://www.cardiogenomics.org/) |
|  | H DCM2 | n=15 DCM patients vs. n=11 normal controls |  |  |  |  |
| [GSE65446](https://www.ncbi.nlm.nih.gov/geo/query/acc.cgi?acc=GSE65446) | H DCM3 | n=6 DCM patients vs. n=4 healthy donors | Ventricular samples | GPL10999  Illumina Genome Analyzer IIx (Homo sapiens) | - 6 patients with medically refractory DCM, excluding specifically those patients with the evidence of ischaemic cardiomyopathy or myocarditis. - 4 ventricular samples were available for mRNA analysis from donor organs used for transplantation (n=2), and donor organs unsuitable for transplantation (n=2). | (4) |

**Table S2. Contractile function data at baseline (pre-MI) and 3 months post-MI revealing significant systolic dysfunction.** Echocardiography parameters were acquired just prior to MI and at 3 months post-MI. Hematology and troponin values at acute phase (3hr and 24hr post-MI) are also shown. Values are Mean±SEM (N=6). P-value from paired t-test. IS - Infarct size; LV – Left Ventricle; EF – Ejection Fraction; ESV - end-systolic volume; EDV - end-diastolic volume; SV -Stroke Volume; HR – Heart rate; CO – Cardiac output; CI – Cardiac index; FAC - Fractional area change; L. strain – longitudinal strain; RBC - red blood cells; HCT – Hematocrit; HGB – hemoglobin.

|  |  | Baseline  (Pre-MI) | Post-MI  (3 months) | P value | Statistical  Significance |
| --- | --- | --- | --- | --- | --- |
|  | **IS % of LV** |  | 13.75±0.62 |  |  |
|  | **LV weight (g)** |  | 66.02±2.47 |  |  |
|  | **Body weight (kg)** | 24.1±1.26 | 26.22±0.90 | 0.010 | * |
| *Echocardiography* | **EF (%)** | 55.03±1.48 | 46.65±1.82 | 0.002 | * |
|  | **ESV (mL)** | 15.75±1.04 | 20.70±1.19 | 0.002 | * |
|  | **EDV (mL)** | 35.32±2.85 | 39.30±3.48 | 0.184 |  |
|  | **SV (mL)** | 19.57±1.93 | 18.60±2.44 | 0.626 |  |
|  | **HR (bpm)** | 134.00±7.64 | 109.50±12.27 | 0.036 | * |
|  | **CO (L/min)** | 2.56±0.17 | 1.94±0.18 | 0.030 | * |
|  | **CI (L/min/m^2^)** | 3.85±0.23 | 2.74±0.21 | 0.025 | * |
|  | **FAC basal (%)** | 46.70±3.02 | 45.13±2.76 | 0.750 |  |
|  | **FAC mid (%)** | 50.42±2.77 | 35.77±5.07 | 0.023 | * |
|  | **FAC apical (%)** | 57.98±2.62 | 27.73±3.61 | 0.001 | * |
|  | **L. strain (%)** | -23.03±1.70 | -17.35±1.00 | 0.006 | * |
| *Hematology* | **RBC** | 5.43±0.47 | 4.57±0.20 | 0.051 |  |
|  | **HCT** | 31.85±2.29 | 26.53±0.66 | 0.059 |  |
|  | **HGB** | 10.58±0.58 | 9.57±0.29 | 0.165 |  |
| *Troponin* | **Troponin (ng/mL)** | 80.05±20.93 | 352.67±68.10  *(3 hrs post-MI)* | 0.003 | * |
|  | **Troponin (ng/mL)** | 80.05±20.93 | 138.67±30.30  *(24 hrs post-MI)* | 0.077 |  |

**Additional Supplemental Tables and supporting information (excel files)**

**Table S3. Differentially expressed genes (DEGs) between all LV tissue areas 3 months post-MI and Naïve control LV tissue.**

**Table S4. Significantly dysregulated canonical pathways and disease functions between non-infarcted LV areas and Naïve control LV tissue determined by Ingenuity Pathways Analysis.**

**Table S5. Dysregulated Metabolites in non-infarcted LV areas comparing to Naïve control LV Tissue.**

**Table S6. RZ vs CTRL DEGs involved in metabolic functions based on IPA functional annotations**

**Table S7. Comparison of expression of pig BZ and RZ DEGs with differentially expressed genes in LV biopsies of ICM and DCM patients.**

**References**

1. Jiye A, Trygg J, Gullberg J, Johansson AI, Jonsson P, Antti H, Marklund SL, Moritz T. Extraction and GC/MS analysis of the human blood plasma metabolome. *Anal Chem* (2005) **77**:8086–8094. doi:10.1021/ac051211v

2. Schauer N, Steinhauser D, Strelkov S, Schomburg D, Allison G, Moritz T, Lundgren K, Roessner-Tunali U, Forbes M, Willmitzer L, et al. GC-MS libraries for the rapid identification of metabolites in complex biological samples. *FEBS Lett* (2005) **579**:1332–7. Available at: https://doi.org/10.1016/j.febslet.2005.01.029

3. Hannenhalli S, Putt ME, Gilmore JM, Wang J, Parmacek MS, Epstein JA, Morrisey EE, Margulies KB, Cappola TP. Transcriptional genomics associates FOX transcription factors with human heart failure. *Circulation* (2006) **114**:1269–1276. doi:10.1161/CIRCULATIONAHA.106.632430

4. Gonzalez-Valdes I, Hidalgo I, Bujarrabal A, Lara-Pezzi E, Padron-Barthe L, Garcia-Pavia P, Gomez P, Redondo JM, Ruiz-Cabello JM, Jimenez-Borreguero LJ, et al. Bmi1 limits dilated cardiomyopathy and heart failure by inhibiting cardiac senescence. *Nat Commun* (2015) **9**:6473.
